# Are Tonkean macaques able to make intuitive statistical inferences?

**DOI:** 10.7717/peerj.21377

**Published:** 2026-06-30

**Authors:** Alice Beaud, Alyzé Detourbet, Sébastien Ballesta, Hélène Meunier

**Affiliations:** 1 Laboratoire de Neurosciences Cognitives et Adaptatives, Université de Strasbourg, Strasbourg, France; 2 Centre de Primatologie, Université de Strasbourg, Niederhausbergen, France

**Keywords:** Intuitive statistics, Proportional reasoning, Probabilistic inference, Decision-making, Numerical cognition, Non-human primates

## Abstract

**Background:**

In a changing environment, making predictions about probabilistic events from few observational facts has ecological relevance for many species. Recent research has shown that preverbal infants, great apes, and capuchins (*Sapajus spp.*) were able to use proportions to predict the nature of a single item randomly sampled from two populations. However, studies involving macaques (*Macaca fascicularis* and *M. mulatta*) brought contradictory results about whether macaques relied on proportions or other choice heuristics to make intuitive inferences. To provide additional information on the evolutionary origins of this skill and overcome methodological concerns from previous studies, we assessed the ability of Tonkean macaques (*M. tonkeana*) to consider proportions in intuitive statistical inferences.

**Methods:**

In a series of eight experimental conditions, 10 Tonkean macaques had to choose which of two jars, that differed in their relative distributions of a preferred and non-preferred food item, conferred a greater chance of obtaining one preferred item. During each test, the experimenter hid in their hands an item drawn pseudo-randomly from each of the two jars for the subject to make its choice.

**Results:**

Our results highlighted both between and within individual variations in their decisional framework. According to experimental conditions, while few individuals relied on proportions to make statistical inferences, most individuals relied on quantities of preferred items or other heuristics to predict the drawing outcome. Altogether, our results brought some evidence that the ability to perform intuitive statistical inferences may be shared amongst primates, but that the underlying high cognitive demands of this capacity may motivate the use of simpler heuristics in some animals’ everyday decisions.

## Introduction

Many living beings evolve in a changing and uncertain environment. For example, we cannot be sure about what the weather will be like, which vegetables will ripen in the garden, or how many cherries will be left to be harvested after a starlings’ raid. The best that we can do is to make some predictions about future events and states of the world based on past experiences or observational facts. Many clouds in the evening suggest a rainy day tomorrow, the green colour of tomatoes gives reason to believe they will not be mature the day after, and the experience of the last few days shows that starlings have eaten roughly 10% of cherries a day.

While being uncertain, the world around us possesses some statistical regularities and is not totally unpredictable. For non-human animals, being able to anticipate events and to track statistical regularities of their environment could play a key role in their survival (Menzel, 1991; Tujague & Janson, 2017). In the wild, foraging activities imply multiple decisions based on uncertain and sparse information. In the framework of the optimal foraging theory, such foraging strategies have been modelled such that energy intake is optimized while cost linked to moving or inspecting food patches is minimized (Pyke, 2019). Indeed, as food sources are often distributed in patches, predicting when and where high quality food is easily available optimizes decisions concerning patch choice, moving between patches, or exploiting a patch, and should thus offer an evolutionary advantage (Kamil, Krebs & Pulliam, 1987; Stephens, 2008; Pyke, 2019). For example, for frugivores, ripe fruits constitute high quality food, but their availability is ephemeral and varies consequently throughout both seasons and tree species (Riley, 2007). In Taï forest, female chimpanzees (*Pan troglodytes*) take into account several cues such as botanical cues, the number of fruit-bearing trees encountered, and trees synchrony level to increase their probability to find ripe fruits (Janmaat, Ban & Boesch, 2013). In the same way, Japanese macaques (*Macaca fuscata*) use an “inspect all strategy” and rely on the synchronicity of reproductive cycle in same fruit species when foraging (Menzel, 1991). Thus, the ability to make inferences about food patch quality from observed information in an uncertain pattern, of newly emerged fruits for example, give a certain advantage in foraging decision making.

When facing such ecological challenges, making inferences (the ability to generate predictions based on limited information; Wang, 2007), about uncertainty involve numerical and logical cognitive capacities. Basic or intuitive forms of statistical inferences have been well studied in human infants, who lack formal knowledge of probabilities and language, as it could play a crucial role in early life (Denison & Xu, 2019). When only scattered information is available, making intuitive inferences enables humans to form representations of states of the world, shape expectations and acquire knowledge without having in-depth experience of the world (Tenenbaum et al., 2011; Sherman, Graves & Turk-Browne, 2020). In intuitive statistical studies, subjects need to form expectations about (or infer) the probable nature of items randomly drawn from populations, based on statistical information provided by the distributions of items in those populations. From the early age of eight-months, preverbal children have been shown to predict an outcome simply based on observed proportions, and vice versa (Téglás et al., 2007; Xu & Garcia, 2008; Xu & Denison, 2009; Denison & Xu, 2010; Denison & Xu, 2014; Denison, Trikutam & Xu, 2014). These multiple studies strongly suggested that children are endowed with pre-established capacities for intuitive inferential reasoning that can be flexibly adapted according to different contexts.

Among non-human animals (hereafter, animals), numerous studies showed that the ability to discriminate quantities was widely shared, from insects (Gatto & Carlesso, 2019), fishes (Agrillo et al., 2009; Lucon-Xiccato et al., 2015), amphibians (Miletto Petrazzini, Bertolucci & Foà, 2018; Lucon-Xiccato, Gatto & Bisazza, 2018) to birds (Rugani, Vallortigara & Regolin, 2014; Nieder, 2018; Stemmler et al., 2018), mammals, including primates (Hanggi, 2003; Cantlon & Brannon, 2006; Schmitt & Fischer, 2011; Schmitt et al., 2013). Numerical abilities in non-human animals have been shown to be ecologically relevant in several daily activities (Nieder, 2018) such as foraging (Addessi, Crescimbene & Visalberghi, 2008; Schmitt & Fischer, 2011), social interactions (McComb, Packer & Pusey, 1994; Wilson, Britton & Franks, 2002; Benson-Amram et al., 2011) or troops movements (Piantadosi & Cantlon, 2017). For example, in hyenas (*Crocuta Crocuta*, Benson-Amram et al., 2011), lionesses (*Panthera leo*, McComb, Packer & Pusey, 1994) and chimpanzees (*Pan troglodytes*, Wilson, Britton & Franks, 2002), when defending their territory, the decision to confront an intruding group depends on the number of individuals in that group. In baboons (*Papio anubis*), the decision of a group to follow a certain direction depends on the number of individuals who commit to it or not (Piantadosi & Cantlon, 2017).

However, for many species, evidence regarding the ability to make intuitive statistical inferences remains limited. In contrast to tasks testing quantities discrimination, intuitive statistical problems require comparing relative quantities, that is to say proportions, rather than absolute quantities. In fact, calculating the probabilities of a particular event, implies to compare the number of possibilities leading to that event, with the total number of possibilities (Bryant & Nunes, 2012). In other words, statistical reasoning implies to discriminate proportions and understand proportions in terms of probabilities. These capacities are considered to be more cognitively demanding than simple numerical abilities, as reasoning about proportions involves comparing second-order relations between different item quantities (Drucker, Rossa & Brannon, 2016). Up to now, several animals such as pigeons, chicks, and some species of primates, have demonstrated the ability to distinguish proportions (Honig & Stewart, 1989; Emmerton, 2001; Vallentin & Nieder, 2008; Rugani, Vallortigara & Regolin, 2015; Drucker, Rossa & Brannon, 2016; Rugani et al., 2016). For example, using two sets of visual stimuli displayed on a touchscreen, two rhesus macaques (*Macaca mulatta*) were trained to choose sets that contained the highest ratio of positive to negative stimuli, regardless of the absolute number of stimuli. When novel ratios were presented, macaques generalized their performance and still chose the higher ratio of positive stimuli. Following Weber’s law, their performance was modulated by the ratio presented and depended on both the ratio of stimuli number and the ratio of stimuli surface (Drucker, Rossa & Brannon, 2016).

Other research showed that many animal species exhibit a sensitivity to probabilistic variations in reward availability (Rosati, 2017; De Petrillo & Rosati, 2021b). For example, studies of non-human primates’ attitudes to risk, when the probabilities of loss are known, have clearly shown that they take probabilistic contingencies into account when making decisions. Yang & Shadlen (2007) demonstrated that rhesus macaques could make operations with probabilities (Yang & Shadlen, 2007). After learning to associate different shapes with different probabilities, the monkeys were presented with four shapes simultaneously. They managed to estimate approximately the probability resulting from the sum of the probabilities represented by the four shapes.

Several animal species, particularly some non-human primates, appear thus able to discriminate proportions and to process probabilistic contingencies. Because making intuitive statistical inferences is considered as an important cognitive capacity that emerges prior to language development, a growing body of research has investigated its evolutionary origins in non-human primates. To investigate whether apes had such statistical inference capacities, Rakoczy et al. (2014) adapted one of the protocols used in infants (Denison & Xu, 2010; Rakoczy et al., 2014). Apes were presented with two transparent buckets containing both a mix of banana pellets (preferred food) and carrot pieces (non-preferred food) in different proportions. The apes had to choose between two hidden items both drawn randomly by the experimenter, one from the favourable population (bucket A containing more bananas than carrots) and the other from the unfavourable population (bucket B containing more carrots than bananas). Across test conditions, item proportions in both buckets varied to control for the use of simple heuristic reasoning. Crucially, in some conditions, absolute and relative quantities were decorrelated (*i.e.,* the favourable population contained less absolute preferred items, hereafter designed as incongruent condition) to control for the use of absolute quantities to make inferences. Results showed that apes were able to correctly predict that their preferred food had more chance to be drawn from the favourable population, relying on food proportions (Rakoczy et al., 2014; Eckert et al., 2018a). Apes were also able to adjust their behaviour according to the random nature or not of the sampling process and the experimenter’s visual access to the population (Eckert et al., 2018b). However, they failed at inferring the distribution of populations, based on a representative sample drawn (Eckert, Rakoczy & Call, 2017). In the same way, they did not understand that a physical constraint (*e.g.*, a barrier that physically blocked the sampling of certain items in a population) could make the sampling process non-random, so that the sample no longer reflected the proportions in the populations (Eckert et al., 2021).

While a consensus seems to have been reached on the great apes’ ability to make statistical inferences from population to sample, the results for other non-human primate species do not seem to be as conclusive. In macaques, two species have been tested with contrasted findings. Long-tailed macaques (*Macaca fascicularis*) failed to draw statistical inferences when absolute and relative quantities of items were incongruent. Except for one successful individual in all conditions, the remaining ten monkeys based their decisions on other heuristics (Placì et al., 2018). To the contrary, these monkeys managed to make predictions under uncertainty when inference was made from relative frequencies of past events (Placì et al., 2019). In another study, rhesus macaques showed some abilities to predict a single future event depending on the favourable or unfavourable proportions of items (De Petrillo & Rosati, 2019b). Monkeys’ responses were measured thanks to a violation-of-expectation paradigm that was used for testing statistical intuition in human infants (Téglás et al., 2007). Although this study suggests intuitive statistical abilities are present in rhesus monkeys, the experimental method employed did not require any explicit choice from individuals, making the results difficult to compare with those obtained in apes (Rakoczy et al., 2014; Eckert et al., 2018a) and long-tailed macaque studies (Placì et al., 2018). In capuchins (*Sapajus spp.*), only one study made by Tecwyn et al. (2017) showed that capuchins were making probabilistic inferences based on relative quantities (Tecwyn et al., 2017). Very recently, intuitive statistical inference abilities were also investigated in small apes (Crimston et al., 2025). Only one siamang (*Symphalangus syndactylus*), among the two tested, succeeded at drawing inferences from populations to samples in the two conditions where absolute and relative quantities of food items were incongruent. The two white-cheeked crested gibbons (*Nomascus leucogenys*) and the remaining siamang made inferences based on relative quantities but only when the outcome were certain (one of the two populations contained 100% of preferred food items). Yet, research on various cognitive tasks involving a choice between different options must rule out the possibility that subjects rely on simpler associative learning rules (*i.e.,* Call, 2022; Canteloup et al., 2017). In both studies with capuchins and small apes, authors could not exclude that individuals have used a simpler heuristic rule such as “avoiding the jar containing the greater number of non-preferred items.”

Overall, both in long-tailed macaque and capuchin studies, group performances were surprisingly low. Success rates, although significant, were only between 60 and 68% at the group level, with chance level being at 50% (Tecwyn et al., 2017; Placì et al., 2018). Lower performances in monkeys compared to apes (71%; Rakoczy et al., 2014) could be explained by lower cognitive capacities as it is the case in some other cognitive domains such as tool use, spatial cognition, or short-term memory (Amici, Aureli & Call, 2010; Schmitt, Pankau & Fischer, 2012) but the ecological relevance of having intuitions about probabilities (De Petrillo & Rosati, 2019a; De Petrillo & Rosati, 2021b) makes this hypothesis less likely. Hence, due to a limited number of studies and a lack of experimental control for simpler heuristic of choice, monkeys’ intuitive inference capacities remain overall poorly understood. Therefore, our study aimed at testing another macaque species, the Tonkean macaque (*Macaca tonkeana*), using a more controlled version of the protocol from Placì et al. (2018) to shed light on the evolutionary history of intuitive statistical capacities among primates. As well as other macaque species tested previously, Tonkean macaques show complex physical and socio-cognitive skills (Joly et al., 2017) as they seem able to attribute perceptions and intentions to others (Canteloup et al., 2016; Canteloup & Meunier, 2017, but see Costes-Thiré et al., 2015; Canteloup et al., 2017; for contrasting results), are capable of tool-use (Bandini & Tennie, 2020) and appear well-aware of social relationships in their group (Whitehouse & Meunier, 2020). Our main goal was to test the existence of statistical inference abilities in Tonkean macaques, and more specifically their ability to predict the nature of a sample based on the nature of the population from which it was randomly drawn. According to recent studies testing these abilities in closely related species (Tecwyn et al., 2017; Placì et al., 2018; Placì et al., 2019; De Petrillo & Rosati, 2019b), we expected Tonkean macaques to possess the ability to make intuitive statistical inferences, at least to be able to make inferences from absolute quantities. By overcoming the previous methodological shortcomings, we predicted higher group performance at the task than previously obtained. Because previous studies showed a high interindividual variability, a second objective was to assess statistical inference abilities at the individual level. Analysing results at group level could obscure the possibility that individuals use different quantity-based heuristics to solve the proposed tasks and we thus aimed to investigate if and how the decisional strategies used to solve the task were different among individuals. These individual differences could also bring more insights into the selected cognitive mechanisms (Thornton & Lukas, 2012; Boogert et al., 2018; Schubiger, Fichtel & Burkart, 2020).

To test their ability to make intuitive statistical inferences, we presented Tonkean macaques with two mixed populations of preferred and non-preferred items to test whether they were able to infer from which population their preferred item had more chance to be randomly sampled. Across different conditions, both the quantities and proportions of items varied to investigate which quantitative features individuals based their choice on. To understand whether individuals were relying on proportions or on other heuristics, such as quantity of preferred or non-preferred items, we adapted some of the experimental conditions conducted in Placì et al. (2018) and added novel experimental conditions. In contrast to previous studies, we controlled for the difference in total quantity of items between populations. Indeed, in a situation with equal proportions, individuals might be more attracted toward the higher number of items, notably preferred items, or rather be biased towards the lowest number of items, where the draw might be considered as more predictable. Thanks to both our multiple conditions and additional methodological improvements, our study aims to understand whether Tonkean macaques have intuitive statistical capacities similarly to other primate species and provide a better comprehension of individuals’ differences in decision-making strategies.

## Materials and methods

Portions of this text were previously published as part of a preprint (https://theses.hal.science/tel-04894777v1).

### Subjects and housing

All individuals that participated in this study were housed at the Primate Center of the University of Strasbourg. Subjects were 23 Tonkean macaques from three different social groups, two male groups (hereafter M1 and M2 groups) and a multi-male, multi-female group (hereafter MF group). M1 and MF groups lived in semi-free-ranging conditions in a wooded enclosure of respectively 1,364 m^2^ and 3,788 m^2^ and had permanent access to an outdoor and an indoor enriched shelter (respectively 21.7 m^3^ and 51.33 m^3^). M2 group lived in an enriched outdoor shelter (105.9 m^3^) with access to an enriched indoor enclosure (32.2 m^3^). M1 was composed of six adult males and M2 of five adult males. MF group included 29 individuals (two adult males, 11 adult females and 16 juveniles). From all groups, 23 individuals participated in the experiment but only 13 individuals passed the training phase and completed at least one test session. All animals were fed with commercial monkey pellets seven days a week and with a supply of fresh fruits and vegetables once a week. Water and food were freely available at all times. All subjects previously participated in cognitive testing involving solid or liquid reward deliveries, but never in tasks testing proportional reasoning. Data were collected from 15/03/2023 to 18/10/2023 (Table 1). Measurements were conducted opportunistically and approved by the ethical committee of the Primate Center of the University of Strasbourg which is authorized to house non-human primates (registration no. B6732636). The research further complied with the EU Directive 2010/63/EU for animal experiments.

**Table 1:** Subjects’ demographic and experimental information. First 13 individuals passed the training phase and completed at least one test session. The maximum number of trials was carried out for the conditions indicated in bold (20 trials). Ten last individuals were not included in analyses as they did not complete any experimental test trials. Two individuals passed the training phase (in bold).

Individual	Short name	Sex	Date of birth	Group	Data collection	Experimental conditions
Abricot	Abr	M	14/05/2013	M2	08/09/2023 − 18/10/2023	**1-2a-2b-3-4-5a-5b-6**
Alaryc	Ala	M	19/03/2013	M2	07/09/2023 − 17/10/2023	**1-2a-2b-3-4-5a-5b-6**
Barnabe	Bar	M	05/12/2014	M2	07/09/2023 − 17/10/2023	**1-2a-2b-3-4-5a-5b-6**
Dory	Dor	F	22/03/2016	MF	15/03/2023 − 15/09/2023	**4-**2b-6
Eric	Eri	M	28/03/2017	MF	15/03/2023 − 13/06/2023	**1-2a-2b-3-4-5a-5b-6**
Ficelle	Fic	F	21/02/2018	MF	15/03/2023 − 26/05/2023	**1-2a-2b-3-4-5a-5b-6**
Horus	Hor	M	28/08/2020	MF	22/03/2023 − 04/10/2023	**1-2a-2b-3-4-5a-5b-6**
Jeanne	Jea	F	14/12/1995	MF	08/08/2023 − 22/09/2023	1-2a-3-5b
Nema	Nem	F	05/01/2011	MF	16/03/2023 − 28/09/2023	**1-2a-2b-3-4-5a-5b-6**
Nereis	Ner	F	15/01/1999	MF	15/03/2023 − 05/05/2023	**1-2a-2b-3-4-5a-5b-6**
Olli	Oll	M	29/11/2010	M1	15/03/2023 − 07/05/2023	**1-2a-2b-3-4-5a-5b-6**
Wallace	Wal	M	29/05/2007	M1	15/03/2023 − 16/08/2023	1-2a-5a-6
Walt	Wat	M	24/10/2007	M1	15/03/2023 − 11/05/2023	**1-2a-2b-3-4-5a-5b-6**
Berenice	Ber	F	13/11/2014	MF	24/04/2023 − 15/09/2023	Training (6 sessions)
Cesar	Ces	M	05/06/2015	M2	08/09/2023 − 09/10/2023	Training (13 sessions)
Gaia	Gai	F	23/10/2019	MF	06/04/2023 − 04/05/2023	Training (6 sessions)
Gandhi	Gan	M	16/04/2019	MF	22/03/2023 − 13/06/2023	Training (7 sessions)
Hercule	Her	M	05/09/2020	MF	21/03/2023 − 18/08/2023	Training (9 sessions)
Iron	Iro	M	30/12/2021	MF	11/08/2023 − 05/09/2023	Training (6 sessions)
Patchouli	Pat	M	17/07/2011	MF	16/03/2023 − 08/08/2023	**Training (9 sessions)**
Yang	Yan	M	10/04/2009	M1	14/03/2023 − 11/04/2023	Training (15 sessions)
Yannick	Yann	M	11/11/2009	M1	15/03/2023 − 27/03/2023	**Training (4 sessions)**
Yin	Yin	F	12/07/2009	MF	16/03/2023 − 27/04/2023	Training (10 sessions)

### Experimental setup

**Figure 1:**
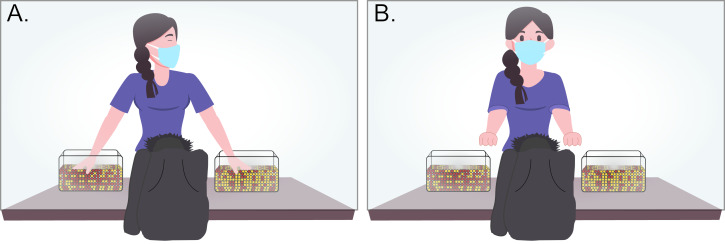
Experimental set-up and drawing procedure. (A) After showing explicitly the content of jars, the experimenter picked at random (eyes closed and head not directed towards the jars), one item from each jar at the same time. (B) The experimenter offered to the subject to choose between two items hidden in the experimenter’s hands. Red items correspond to non-preferred food items and yellow items to preferred food items. (Use of adapted vector images designed by Freepik).

Subjects of M1 and MF groups were tested individually by voluntarily joining an experimental room (4 m × 3 m × 2.5 m) adjacent to their outdoor enclosure. Subjects of M2 group were tested in their enriched outdoor shelter. The set up consisted of a tray (89 cm × 56 cm) put onto a table (115 cm × 88 cm) on the experimenter’s side, separated from the subject area by a wire mesh. During the test, the subject sat on a perch at table height (Fig. 1). Two rectangular transparent plastic jars, each measuring 20 cm × 9 cm × 13 cm, were presented simultaneously. Each jar contained a mix of preferred and non-preferred food items.

### Procedure

#### Food items

The preferred food chosen was an unsalted half peanut (hereafter peanut) and the non-preferred food was a raw kidney bean (hereafter bean). The two types of food matched different constraints as they were of comparable shape, volume and of different colours (light beige and dark red) so that the items were clearly distinguishable visually. In addition, these food items were non-perishable which eases these kinds of experimental protocols.

#### Preference test

Individuals’ preferred (peanut) and non-preferred (bean) food items were determined by a preference test. The different food items, of equivalent size, were presented in pairs. The test consisted of four sessions of 12 trials, with one session per day per individual. On each trial, a peanut and a bean were presented simultaneously in the experimenter’s hands, equidistant from the individual. When the individual pointed to the chosen item, it was immediately given as a reward and the second withdrawn. An individual was considered to have a constant preference for the peanut over time if it chose it on at least 10 out of 12 trials in three consecutive sessions.

Moreover, to check for the consistency of the individuals’ preference for one of the two items, we conducted two preference trials before each subsequent experimental session. If the individual chose the preferred item in both of these trials, we moved on with the session. If the individual chose the preferred item only one time among the two trials, we conducted two additional preference trials. The individual then needed to select the preferred item in at least 3/4 trials to proceed with the session. If it did not, the session was postponed.

Although there was a high preference for peanuts against beans, some individuals were not sufficiently motivated by obtaining a peanut or got less motivated as the number of sessions increased. Thus, for some individuals listed, to maintain a proper level of motivation of the subject to perform the task, each time they obtained a peanut they also obtained a dried grape (Table S1). From the moment these individuals started to get a dried grape in addition to a peanut, we stuck to this procedure until the end of the experimental protocol for these subjects. Adding a dried grape did not have any influence on overall performance (for more details see Fig. S1 and Table S2).

#### Procedure for the statistical inference task

Individuals were presented with one or two sessions a day. All sessions were carried out by one experimenter and one assistant, whose role alternated.

At the beginning of each trial, the experimenter presented two transparent jars (except in the olfactory control condition, see Table 2), each containing a mixture of preferred and non-preferred food items in different proportions. Each time the contents of the jars changed, the experimenter presented the contents of the jars at the beginning of the session. The experimenter shook each jar and tilted it forward to increase the visibility of items. The experimenter always started with the jar to her right. The experimenter then turned both jars simultaneously through 360° so that the individual had a visual access to the entire circumference of the jar, and simultaneously shook and tilted them forward to show the contents of the jars once again. At each trial, the experimenter pretended to draw one item of each jar. The nature of the item to be drawn was predetermined in a pseudo-probabilistic manner to ensure that the succession of items drawn reflected the proportions in jars. Thus, at the start of each trial, the assistant subtly placed (hidden from the monkey’s view) an item of food in each of the experimenter’s hands. The experimenter then placed her hands in the jars (pretending to have empty hands), closed her eyes and ostensibly positioned himself so as not to see the contents of the jars. The position, head and eyes orientation taken by the experimenter varied with each trial in a random order (Fig. S2) to make sure that no associative learning was possible between a specific position and a given outcome. The posture and direction of the experimenter’s gaze made items appear to be picked at random, as the experimenter had no visual access to the contents of the jars (Canteloup, Bovet & Meunier, 2015a; Canteloup, Bovet & Meunier, 2015b; Eckert et al., 2018b). After adopting a posture, the experimenter stirred the contents of the jar three times and closed her fists before advancing them towards the individual. After touching one of the two hands, the individual received the item in the chosen hand as reward. The unchosen item was not revealed to the individual because this additional information could have distracted the individual, influenced its choice on subsequent trials, or increased probability of learning (for comparable protocol see Placì et al., 2018; but see also Tecwyn et al., 2017 for an alternative protocol). On half of the trials, the experimenter’s arms were crossed after they were advanced towards the individual. This was made to ensure the individual was choosing between the two samples and not just choosing the side where favourable population was located (the transparent jars that were visible at the time of the choice). Trials with crossed hands were defined randomly within each session.

**Table 2:** Summary of the eight experimental conditions tested. The jars are depicted in side view as black rectangles with a white background when transparent, and with grey background when opaque (condition 6 only). The yellow circles inside the jars represent the preferred food (half peanut), the red circles represent the nonpreferred food (raw kidney bean). Each quantity is written below each jar such as the first number is peanuts and the second beans (peanut : bean). Jar A is always the favourable jar or the lowest quantity jar in control conditions. In congruent conditions, choosing the favourable population of peanuts correlates with choosing the highest quantity of peanuts, while in incongruent conditions, it is not. In bold are emphasised the favourable proportions. Each condition shows some specificities concerning the number (n) of peanuts, beans, and total items. In condition 1, items proportions between jars are reversed and total quantity of items is the same. In condition 2a and 2b, peanut quantity is higher in unfavourable jar while it is lower in favourable jar. In condition 3, peanut quantities are the same in both jars while proportions vary. In condition 4, bean quantities are the same in both jars while proportions vary. In condition 5a and 5b, proportions are the same in both jars (50/50) but total items quantity varies. In condition 6, item proportions are reversed but jars are opaque for olfactory control. The last three columns show expected behavioural responses in each condition according to the decisional strategy used, *i.e*. reliance on proportions (ROR strategy), reliance on peanut quantities (RQP strategy) or reliance on both peanut quantities and proportions (ROR and RQP strategy) to make inferences. A cross ‘X’ beneath a jar indicates that this jar is expected to be chosen more often than chance in the given condition. A cross beneath both jars of the same condition indicates that both jars are expected to be chosen with equal frequency. A cross between brackets ‘(X)’ beneath both jars of the same condition indicates that one or the other jar is expected to be chosen more often than chance. As described in the main text, ROR is for “ratio of ratio” and RQP is for “ratio of peanut quantities”.

**Conditions numbers**	**1**		**2a**		**2b**		**3**		**4**		**5a**		**5b**		**6**	
**Conditions labels**	Quantity-proportion congruent		Quantity-proportion incongruent		Quantity-proportion incongruent		Ctrl-preferred item quantity incongruent		Ctrl-non preferred item quantity congruent		Quantity-Ctrl		Quantity-Ctrl		Olfactory-Ctrl	
**Jar**	Jar A	Jar B	Jar A	Jar B	Jar A	Jar B	Jar A	Jar B	Jar A	Jar B	Jar A	Jar B	Jar A	Jar B	Jar A	Jar B
**Symbol**	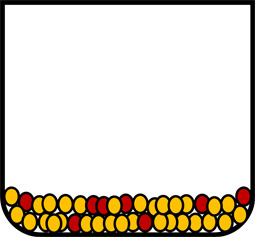 **240: 60**	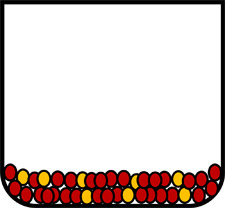 60: 240	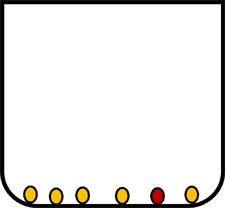 **32: 8**	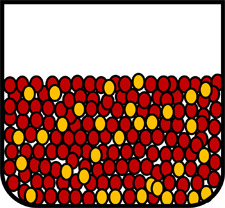 256:1024	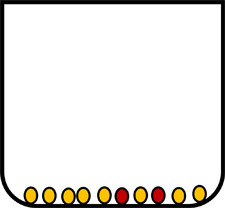 **56: 14**	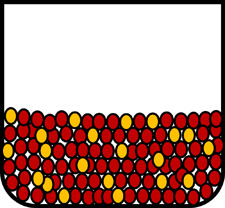 168: 672	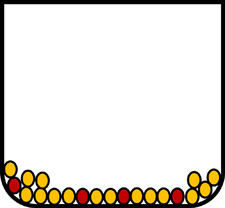 **128: 32**	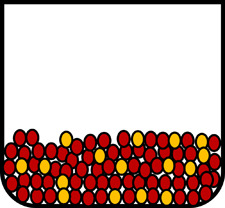 128: 512	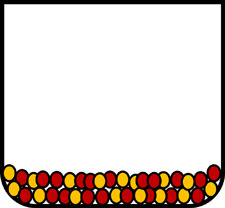 **128: 160**	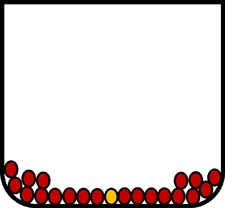 8: 160	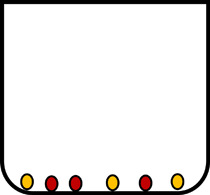 20: 20	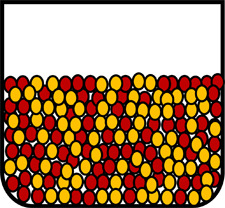 640: 640	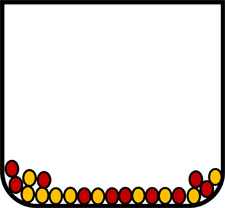 80: 80	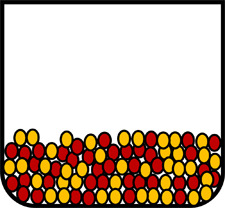 320: 320	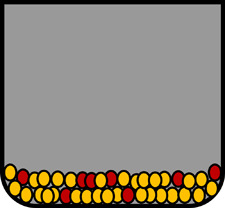 **240: 60**	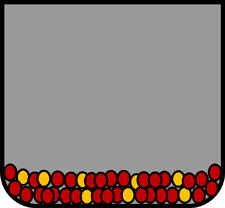 60: 240
**n peanuts**	240	60	32	256	56	168	128	128	128	8	20	640	80	320	240	60
**n beans**	60	240	8	1024	14	672	32	512	160	160	20	640	80	320	60	240
**n total**	300	300	40	1280	70	840	160	640	288	168	40	1280	160	640	300	300
**RQP**	0,20		0,11		0,25		0,50		0,06		0,03		0,20		Non visible	
**ROR**	16		16		16		16		16		1		1		Non visible	
**ROR strategy hypothesis**	**X**		**X**		**X**		**X**		**X**		**X**	**X**	**X**	**X**	**X**	**X**
**RQP strategy hypothesis**	**X**			**X**		**X**	**X**	**X**	**X**			**X**		**X**	**X**	**X**
**ROR/RQP strategy hypothesis**	**X**			**X**	**(X)**	**(X)**	**X**		**X**			**X**	**(X)**	**(X)**	**X**	**X**

Every session was videotaped. During the experimental sessions, the experimenter and assistant were coding individuals’ choices. The assistant recorded on a paper sheet a success when the favourable or the emptiest jar was chosen, and a failure otherwise. A naïve observer coded independently 35 videos which included 20% of the sessions randomly selected. Cohen’s kappa was 0.99 (*p*-value < 0.01) for whether the subject selected the experimenter’s hand containing the item from jar on the right or on the left. Disagreements were resolved through discussion.

##### Training condition.

The training condition was first presented to all individuals to train them for the experimental procedure. Each jar contained a population made up of 100% of the same food item (one jar full of peanuts and the other full of beans) and each contained 300 items. In this condition, no use of item proportions was required to choose the sample drawn. One session consisted of two preference trials, to check for the consistency of the individuals’ preference for one of the two items, and ten subsequent training trials. To be tested in experimental conditions, individuals needed to validate the training condition with 8/10 correct answers during three consecutive sessions. When individuals developed a side-bias during the training period, we conducted two *visible-item* sessions. An individual was considered as side-biased if it chose the same side in at least 8/10 trials or if it chose the same side in all of the crossed-arms trials of a session. A *visible-item* session was identical to a training session except that both drawn items were immediately visible to the individual after the drawing and at the moment of the choice instead of being hidden in the hand. If no mistake was made during the two visible-item sessions, the subject returned to the classic training session (Fig. S3). Among the 13 individuals that passed the training phase, three of them received *visible-items* sessions (*i.e.,* two for *Abricot*; two for *Ficelle*; four for *Horus*).

##### Experimental conditions.

Subjects participated in eight conditions in which proportions of preferred and non-preferred food contained in each jar varied (Table 2). Two sessions of ten trials were conducted in each condition. All test conditions (and sessions) were presented in a random order for each individual, to minimise possible reinforcement learning across sessions. Each session began with two preference trials, four training trials, and then ten test trials. The four training trials (which included two crossed-arms trials) were conducted to ensure that individuals were sufficiently motivated and did not show obvious side biases in their choices (as previously defined). To continue with the test trials, individuals had to make no error over the four training trials. If the individual made a maximum of one error over the four training trials and had no side-bias in the previous session, it needed to make no error in four more training trials, to move on with the test trials. If the individual made at least one mistake over the four training trials and had a side-bias in the previous session, no test trials were conducted. The individual kept going with the process to remove its bias. Two visible-item sessions were conducted before moving back to training sessions. The individual then needed to make at least 8/10 correct answers in two consecutive training sessions, to come back in the test sessions process. In every other case, test trials were delayed (Fig. S4).

###### Condition 1: quantities and proportions congruent.

In this test condition, the aim was to see whether individuals could make an inference about the nature of a sample from proportions that were simply reversed and when the total number of items in each jar was identical. Jar A contained a majority of peanuts relative to the number of beans (peanuts: beans, 240: 60) and jar B contained the opposite proportion (60: 240).

###### Condition 2: quantities and proportions of preferred items incongruent.

The aim of condition 2 was to test whether individuals were choosing from proportions or choosing from quantities. Indeed, in condition 1, the proportion of preferred items was congruent with their quantity. In jar A, the ratio of peanuts was higher than in jar B, but there were also more peanuts (in quantity) in jar A (240) than in jar B (60). In condition 2, the absolute and relative quantities of each item were incongruent, and the total number of items in each jar was different. Two sub-conditions were designed that differed in total quantity discrepancy between two jars. In condition 2a, jar A contained a favourable proportion but eight times less quantity of peanuts than jar B (peanuts: beans, 32: 8) and jar B contained an unfavourable proportion with a higher quantity of peanuts (256: 1024). In condition 2b, jar A contained a favourable proportion but three times less of peanuts than jar B (peanuts: beans, 56: 14) and jar B contained an unfavourable proportion with a higher quantity of peanuts (168: 672).

###### Condition 3: control for preferred item quantity—incongruent.

A third condition tested individuals’ ability to rely on proportions while limiting the effect of a strong inhibitory control demand for the preferred reward. Indeed, in condition 2, jar B contained many more preferred items (256 and 168) in quantity than jar A (32 and 56), which may have encouraged individuals to prefer the sample from jar B despite an unfavourable ratio of peanuts. In this condition, the quantity of preferred food in each jar was identical, but the quantity of the non-preferred items varied. We thus had jar A with a favourable proportion of preferred items (peanuts: beans, 128:32) and jar B with an unfavourable proportion of preferred items (peanuts: beans, 128:512).

###### Condition 4: control for non-preferred item quantity—congruent.

Condition 4 was designed to test whether individuals did not rely on a simple alternative learning rule. Indeed, individuals could solve all previous conditions by always avoiding the jar containing the most non-preferred items. To test this hypothesis, each jar contained the same quantity of non-preferred items, while the quantity of preferred items varied. Thus, jar A contained a majority of preferred items (peanuts: beans, 128:160) and jar B contained a majority of non-preferred items (peanuts: beans, 8:160).

###### Condition 5: control for total item quantity variations.

Condition 5 was an additional control condition to test whether individuals’ choices could be biased by the total quantities of items present in the jars. Indeed, individuals could establish strategies based on their preference for the fullest or least full jar. Moreover, this condition would also test how much the discrepancy in peanut quantities could drive decision making in previous conditions 2 and 4. In this condition, both populations had the same proportions of preferred and non-preferred items (50% peanut and 50% beans), but the total quantities in each jar differed according to two sub-conditions. In condition 5a, jar A contained 40 items and jar B a total of 1,280 items. This made it possible to mimic a quantity discrepancy similar to that in condition 2a, where all items in jar A were visible (no hidden items in the jar), unlike jar B. In condition 5b, jar A contained 160 items and jar B a total of 640 items. This made it possible to mimic a quantity discrepancy similar to that in condition 3, where the two jars contained items hidden by each other but in different quantities.

###### Condition 6: olfactory control.

Condition 6 controlled the use of olfactory cues by individuals. To test whether individuals could guess the nature of the drawn reward hidden in the experimenter’s fists by their sense of smell, condition 1 was repeated using two opaque jars. Each jar had a mixed composition of preferred and non-preferred food, with the ratio reversed (jar A, 240:60; jar B, 60:240).

Experimental conditions can thus be classified into three categories relative to the relation between the quantities and proportions of preferred items. The congruent conditions include conditions 1 and 4, where the proportions and the quantities of peanuts in both jars varied in the same way and were confounded with each other. Theoretically, both comparison of peanut proportions and/or peanut quantities could thus be used to solve the task. The incongruent conditions include conditions 2a, 2b and 3, where peanut quantities and proportions were disentangled. Thus, in these conditions, the task could be solved by using peanut proportions and/or bean quantities. The control conditions include conditions 5a, 5b, and 6. In conditions 5a and 5b, peanuts proportions were the same in both jars, but peanut quantities varied. In condition 6, monkeys were not able to see which proportions nor quantities were in the jars.

Except for control conditions, one jar always contained a higher proportion of preferred item (jar A) than the other jar. The gap separating the proportions of the two populations could be defined by a ratio of ratios (ROR): the ratio between the proportion of preferred items in the favourable population and the proportion of preferred items in the unfavourable population (Eckert et al., 2018a). The ROR remained constant under all these conditions, that is 16.

\begin{eqnarray*}ROR= \frac{Ratio~of~peanuts~to~beans~in~the~more~favorable~population}{Ratio~of~peanuts~to~beans~in~the~less~favorable~population} . \end{eqnarray*}

##### Sampling and rewarding specificities.

In all conditions, due to the relatively small number of trials, the items were drawn in a pseudo-probabilistic manner to ensure that the drawn items unambiguously reflected the proportions of peanuts and beans present in each jar. For all conditions, two lists of items to be drawn, one for the favourable jar and another for the unfavourable one, were predetermined. In congruent and incongruent conditions except for condition 4, eight peanuts and two beans of the ten trials were listed for the favourable jar (jar A), while two peanuts and eight beans were listed for the unfavourable jar (jar B). The order in which the items were listed was pseudo-randomised, with the following constraints: (1) the first four items were the majority items. (2) the fifth item was the minority item. (3) the last item was always the majority item. (4) there were never two minority items in a row. The order of the items drawn was thus determined for each jar independently to reflect the independency of the two simultaneous samplings. The first item drawn for either jar A or B matched to the individual’s first choice for either jar A or B. Thus, if the individual chose jar B for the first time on the fifth trial, it would receive a bean, as this was the first item in the order established for jar B.

To coincide with the different ratios in condition 4, four peanuts out of the ten first trials and five peanuts out of the next ten trials (second session) were drawn from jar A in a pseudo-randomized order with the constraints that the first item was a peanut and no more than two beans in a row were selected. Also, only one peanut was drawn randomly from jar B over the two sessions.

In control conditions (conditions 5a and 5b), food items were listed and drawn in a predetermined and pseudo-random order, so that no more than two identical items were drawn in a row from the same jar. Of the ten trials, as many peanuts as beans were listed to match the identical proportions of food items in the two jars.

### Data analyses

#### General statistics

All statistical analyses were conducted in R (RStudio version 4.3.2 2023) with the packages “lmerTest” version 3.1-3 (Kuznetsova, Brockhoff & Christensen, 2017), “lme4” version 1.1-35.1 (Bates et al., 2015), “car” version 3.1-2 (Fox & Weisberg, 2019), “gmodels” version 2.18.1.1, “ggplot2” version 3.4.4 (Wickham, 2009), “DHARMa” version 0.4.6.

To test whether the median group performance for each condition was different from chance, we used a two-tailed Wilcoxon signed-rank test.

We investigated whether monkeys were relying on peanut proportions, peanut quantities, or bean quantities in their response to the task. According to Weber’s law, the difference in quantities or proportions between two jars of a same condition was expressed by a ratio of quantities or proportions (Table S3). In the following sections, we will use the notations below for the different variables tested:

The ratio of quantities of peanuts (RQP) between two jars of a same condition:

\begin{eqnarray*}RQP= \frac{Peanut~quantity~in~right~jar}{Peanut~quantity~in~right~jar+Peanut~quantity~in~left~jar} . \end{eqnarray*}

The ratio of quantities of beans (RQB) between two jars of a same condition:

\begin{eqnarray*}RQB= \frac{Bean~quantity~in~right~jar}{Bean~quantity~in~right~jar+Bean~quantity~in~left~jar} . \end{eqnarray*}

The ratio of proportions (ratio of ratio) of peanuts between two jars of a same condition:

\begin{eqnarray*}ROR= \frac{Ratio~of~peanuts~to~beans~in~the~right~jar}{Ratio~of~peanuts~to~beans~in~the~left~jar} . \end{eqnarray*}

We ran a Generalized Linear Mixed Models (GLMM) with a binomial distribution and a logit link function to evaluate on which food ratio monkeys relied on to make their choice, the impact of experimental factors other than food items quantities that varied during the procedure on monkeys’ performance, and to evaluate the effect of conducting two sessions in each condition on monkeys’ choice:

*Model (B)* :

Choice for the jar on the right ∼logRQP + logROR + state of hands + experimenter identity + jar location + session + (1—individual identity) + (1—experimenter position).

We put as fixed variable the jar (right or left) selected by the individual and as explanatory variables the logarithm of ROR and RQP, the states of hand (crossed or not crossed), the experimenter identity, the location of the jar A and the session (first or second session in one condition). The individual identity and the experimenter position (Fig. S2) were put as random variables. This model was selected as the model that fit the best the group data based on the “Variance Inflated Factor” (VIF) and the Bayesian Information Criterion (BIC) of the model (Table S4; Galvão & Araújo, 2009). BIC is a parameter more restrictive towards the number of variables and allow to select the best model with the least number of variables which could help with correlation and collinearity problems (Lebarbier & Mary-Huard, 2006).

#### Individual decisional strategies analyses

We first analysed individuals’ performance in each condition by running two-tailed binomial tests, to calculate whether the probability to observe the number of successes was different from chance with a total of *n* = 20 trials and a probability of success of *p* = 0.5 for each trial. This analysis had the advantage of highlighting both inter-individual variations and within individual variations across conditions, while allowing a straightforward interpretation, condition-by-condition. However, some ambiguous individual responses may be difficult to evaluate with such statistics.

We therefore conducted a secondary analysis to examine individuals’ quantity-based or proportion-based decisional strategies. This analysis took into account all quantitative parameters (RQP, peanut quantities; RQB, bean quantities; and ROR, proportions) varying across conditions and combined individuals’ responses across all conditions. To this end, the following Generalized Linear Models (GLM) models with a binomial distribution and a logit link function were compared according to their BIC for each individual:

*Model (1)* Choice for the jar on the right ∼logRQP + logRQB + logROR.

*Model (2)* Choice for the jar on the right ∼logRQP + logRQB.

*Model (3)* Choice for the jar on the right ∼logRQP + logROR.

*Model (4)* Choice for the jar on the right ∼logRQB + logROR.

*Model (5)* Choice for the jar on the right ∼logRQP.

*Model (6)* Choice for the jar on the right ∼logRQB.

*Model (7)* Choice for the jar on the right ∼logROR.

We made a model selection to identify which model fits the best the behaviour of each individual. We considered the best model to be the one with the lowest BIC, which differed by at least two points from the other models. A difference of BIC lower than two between two models does not indicate that one model fits significantly the data better than the other (Raftery, 1995; Raftery, 1999). A key limitation of this analysis was that however, the factor ROR varied much less (three values) than the RQB and RQP factors. Therefore, we interpreted our results of this latter analysis in light of the individual binomial statistics, made condition-by-condition.

To refine our analysis, we also investigated whether the nature of the food item received in one trial could affect monkeys’ choice in the next trial. The commonly “Win-Stay/Lose-Shift” (WS/LS) strategy have been shown to affect probabilistic choice in monkeys (Ferrari-Toniolo, Bujold & Schultz, 2019). In our design, monkeys may experience different reward outcomes depending on their previous choice, as reward outcome were designed to reflect population distributions. We thus ran a GLMM with a binomial distribution and a logit link function on data of each individual across all conditions. We put as fixed variable the choice to stay or shift and as explanatory variable, the food item obtained by the subject in the previous trial. The session was put as a random variable.

## Results

### Preference test

All 13 individuals included in the study, selected the peanut compared to the bean in 12/12 trials in three consecutive sessions (except for one individual *Barnabé*, which did one of the three sessions with the score of 11/12).

### Training condition

On average, individuals validated the training condition in six sessions of ten trials. The minimum number of sessions to validate was three and the maximum was ten.

### Intuitive statistical inference task

#### Group performance

**Figure 2:**
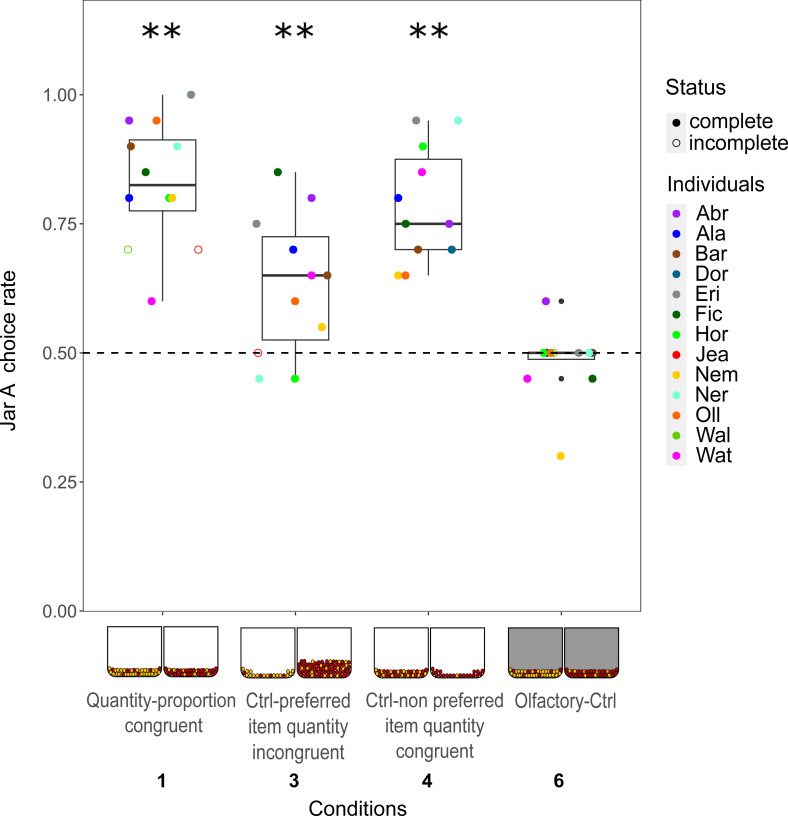
Group performance across conditions testing inference based on proportions. The proportion of trials in which individuals selected the sample drawn from jar A (favourable population). The dark line in whisker box corresponds to the median. Dash line represents the chance level. Dots represent the individual data according to colours. Status indicates if individuals did 20 trials in the condition (complete status) or less (incomplete status). Total number of trials in each condition was different (*n* = 220 in conditions 1, 4 and 6; *n* = 210 in condition 3). The order in which individuals completed conditions was randomised. ** *p*-value ≤ 0.01 in a two-tailed Wilcoxon signed rank-test.

**Figure 3:**
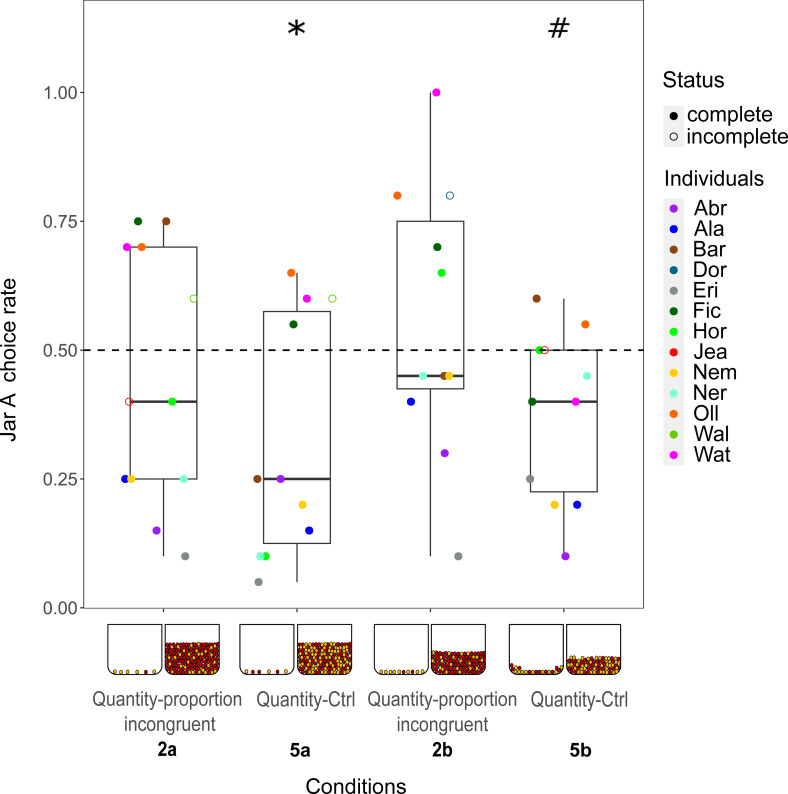
Group performance across conditions revealing inference based on preferred item quantities. The proportion of trials in which individuals selected the sample drawn from jar A (favourable population or lowest quantity population). Incongruent conditions 2 (proportions and quantities are decorrelated) and control conditions 5 are presented. The dark line in whisker box corresponds to the median. Dash line represents the chance level. Dots represent the individual data according to colours. Status indicates if individuals did 20 trials in the condition (complete status) or less (incomplete status). Total number of trials in each condition was different (*n* = 220 in conditions 2a; *n* = 210 in conditions 2b and 5b; *n* = 205 in condition 5a). The order in which individuals completed conditions was randomised. *p-value <0.05 and #*p*-value<0.1 in a two-tailed Wilcoxon signed rank-test.

We analysed the results of 1,715 trials from 13 individuals. Ten individuals completed 20 trials in each of the eight conditions (status complete, filled circle, Figs. 2 and 3) while three individuals did 35 to 40 trials overall (status incomplete, empty circle, Figs. 2 and 3).

For each experimental condition, the favourable jar selection rate (or the least items jar) was compared to the chance level of 50%. In condition 1, when proportions were simply reversed, individuals chose the hand from the favourable population in 84% of the trials, significantly more often than chance (Fig. 2; Wilcoxon signed-rank test, *V* = 78, *n* = 12, *P* = 0.002). Similarly, when bean quantities stayed constant between jars, but proportions differed (condition 4), individuals chose the hand from the favourable population in 79% of the trials, significantly more often than chance (Fig. 2; Wilcoxon signed-rank test, *V* = 66, *n* = 11, *P* = 0.004).

Thus, in congruent conditions, individuals chose significantly more the sample drawn from the favourable population. Also, in condition 3, when quantities and proportions were incongruent, but peanut quantities were the same in both jars, individuals chose the hand from the favourable population in 64% of the trials, significantly more often than chance (Fig. 2; Wilcoxon signed-rank test, *V* = 52, *n* = 11, *P* = 0.01).

Interestingly, when comparing the performance in the conditions 1, 3 and 4, the group succeeded significantly better in choosing the sample drawn from the favourable jar when peanut quantities were varying between jars (congruent conditions 1 and 4) than when they were the same in both jars (incongruent condition 3, Wilcoxon signed-rank test—Conditions 1 *vs* 3: *V* = 54, *n* = 10, *P* = 0.008; Conditions 4 *vs* 3: *V* = 7, *n* = 10, *P* = 0.04).

However, in incongruent conditions 2, when quantities and proportions were incongruent but peanut quantities varied (conditions 2), individuals chose the hand from the favourable jar in 43% of trials (condition 2a) and 54% of the trials (condition 2b), which was not significantly different from chance level (Fig. 3; Wilcoxon signed-rank test, condition 2a, *V* = 27, *n* = 12, *P* = 0.36 ; condition 2b, *V* = 39, *n* = 11, *P* = 0.62). In control conditions, when item proportions did not differ between jars but only total quantities varied (conditions 5a and 5b), individuals chose the jar with the lowest total quantity of items in 30% of the trials (condition 5a) and in 37% of the trials (condition 5b), which tended to be less than chance (Fig. 3; Wilcoxon signed-rank test, condition 5a, *V* = 10, *n* = 11, P = 0.05 ; condition 5b, *V* = 6, n = 11, *P* = 0.056).

In the olfactory control condition, individuals had no visual access to the content of jars and chose the hand from the favourable population in half of the trials (48%), no different from chance (Fig. 2; Wilcoxon signed-rank test, *V* = 3, *n* = 12, *P* = 0.58).

In all conditions (excluded olfactory control condition), GLMM analyse (*Model (B)*) revealed a significant influence of peanut quantities (logRQP; estimate ± s.e. = 0.66 ± 0.06, *z* = 10.8, *p* < 0.01) and proportions (logROR; estimate ± s.e. = 0.51 ± 0.06, *z* = 9.04, *p* < 0.01) on monkeys’ choice. Bean quantities (RQB) did not seem to affect significantly their choice since this variable was not retained in the best model selected (Table S4). Our results indicated that there was no main effect of whether hands were crossed or not (estimate ± s.e. = 0.02 ± 0.11, *z* = 0.16, *p* = 0.87), the experimenter identity (estimate ± s.e. = 0.18 ± 0.14, z = 1.29, *p* = 0.2), and the location of jar A (estimate ± s.e. = −0.05 ± 0.11, z = −0.42, *p* = 0.68). No effect of session was detected (estimate ± s.e. = 0.05 ± 0.12, *z* = 0.47, *p* = 0.64). No significant learning effect of sessions nor trials was thus detected (see also Fig. S5). First-trial performance analyses in each condition also corroborated the group’s findings (Table S6).

#### Individual decisional strategies

To obtain additional information about the inference-based decision-making strategies, *i.e.,* whether individuals used the quantities or proportions to make inferences, we analysed individual data (Table 3 and Table S5). Binomial tests used for each condition highlighted some inter-individual variations in performance (Table 3). Individual responses can be compared to the hypothetical outcomes associated with decisional strategies in Table 2. The subject *Ficelle* chose the sample from the favourable population significantly more than chance in all conditions where proportions differed (but a tendency for condition 2b) and chose at chance when proportions were the same, which could strongly indicate a reliance on proportions (Table 3). *Abricot* and *Eric* seemed to rely either on preferred item quantities or proportions depending on experimental conditions. They chose significantly more the sample drawn from the favourable jar in congruent conditions (conditions 1 and 4) and in incongruent condition 3, where preferred item quantities were equal in both jars. But they chose significantly more the jar with the highest quantity of peanuts in incongruent conditions 2 and control conditions 5 (Table 3). *Alaryc*, *Horus*, *Nereis* and *Nema* seemed to mostly consider preferred item quantities. These individuals chose the sample from the favourable jar significantly more than chance in congruent conditions (except for *Nema* in condition 4). But they chose at chance or significantly more the sample from the jar containing most preferred item quantities in incongruent and control conditions (Table 3). At last, *Barnabé*, *Walt* and *Olli*, showed mixed results that could not be related to any of the strategies described in Table 2.

**Table 3:** Individual (*n* = 10) performances across conditions. Each number represents the choice rate of jar A (favourable or lowest total quantity population) for each individual during the two sessions in each condition. Binomial tests used for each condition highlighted some inter-individual variations. Bold values indicate significant performance.

**Conditions name**	**1**	**2a**	**2b**	**3**	**4**	**5a**	**5b**	**6**
**Quantity/Proportion relation**	Congruent	Incongruent	Incongruent	Incongruent	Congruent	Control	Control	Non visible
**Individuals**								
Abricot	0.95^**^	0.15^**^	0.30	0.80^**^	0.75^*^	0.25^*^	0.10^**^	0.60
Alaryc	0.80^**^	0.25^*^	0.40	0.70	0.80^**^	0.15^**^	0.20^**^	0.50
Barnabe	0.90^**^	0.75^*^	0.45	0.65	0.70	0.25^*^	0.60	0.50
Eric	1.00^**^	0.10^**^	0.10^**^	0.75^*^	0.95^**^	0.05^**^	0.25^*^	0.50
Ficelle	0.85^**^	0.75^*^	0.70	0.85^**^	0.75^*^	0.55	0.40	0.45
Horus	0.80^**^	0.40	0.65	0.45	0.90^**^	0.10^**^	0.50	0.50
Nema	0.80^**^	0.25^*^	0.45	0.55	0.65	0.20^**^	0.20^**^	0.30
Nereis	0.90^**^	0.25^*^	0.45	0.45	0.95^**^	0.10^**^	0.45	0.50
Olli	0.95^**^	0.70	0.80^**^	0.60	0.65	0.65	0.55	0.50
Walt	0.60	0.70	1.00^**^	0.65	0.85^**^	0.60	0.40	0.45

**Notes.**

** *p*-value < 0.01 in a two-tailed binomial test.

* *p*-value < 0.05 in a two-tailed binomial test.

**Table 4:** Individual Win-Stay/Lose-Shift strategy results. The model testing the Win-Stay/Lose-Shift strategy was run on data from each individual across all conditions. Results show the effect of obtaining a preferred food item on the subsequent choice (stay or switch). Bold values indicate significant effects with *p*-values <0.05.

**Predictors**	**Intercept**			**Preferred food in previous trial**		
Individuals	Estimates	*SE*	*p*	Estimates	*SE*	*p*
**Abricot**	0,474	0,241	0,049	0,261	0,350	0,455
**Alaryc**	0,141	0,238	0,553	0,392	0,340	0,249
**Barnabe**	−0,172	0,240	0,474	**0,726**	**0,340**	**0,033**
**Eric**	1,010	0,330	0,002	0,214	0,389	0,583
**Ficelle**	−0,065	0,254	0,800	0,562	0,341	0,099
**Horus**	0,262	0,243	0,280	0,143	0,338	0,672
**Nema**	0,187	0,232	0,420	−0,100	0,334	0,765
**Nereis**	0,173	0,264	0,512	0,101	0,337	0,765
**Olli**	−0,405	0,253	0,109	**0,686**	**0,340**	**0,044**
**Walt**	0,431	0,252	0,087	0,262	0,348	0,451

Our secondary analysis aimed to determine which main quantity parameter influenced monkeys’ decision across all conditions (Table S5). We ran GLMs across all conditions (excluding olfactory control condition) and we selected the model that best fit each individual’s behaviour. The analysis revealed that performance was significantly influenced by peanut proportions (ROR) for two individuals (*i.e., Ficelle* and *Walt*; Table S5). Both peanut quantities (RQP) and proportions (ROR) significantly influenced the performance of one individual (*i.e., Horus*; Table S5). For five individuals, peanut quantities (RQP) significantly affected performance, but peanut proportions (ROR) and bean quantities (RQB) had no effect (*i.e., Alaryc*, *Abricot*, *Eric*, *Nereis*, *Nema*; Table S5). Importantly, none of the subjects mainly relied on non-preferred item quantities to solve the task.

We also analysed the effect of the previous reward outcome on the monkeys’ choice (Table 4). The analysis showed that two individuals significantly used a Win-Stay/Lose-Shift (WS/LS) strategy to solve the task across all conditions (*i.e., Barnabé* and *Olli*).

## Discussion

In the present study, we provided Tonkean macaques with two visible populations composed of a mix of preferred (peanuts) and non-preferred (beans) food items in different proportions. We tested their ability to rely on proportions to predict the nature of a single-item randomly drawn from these two populations. Our findings showed that, at group level, monkeys chose the sample from the favourable population in a high majority of trials, when proportions were reversed between the two populations (condition 1, Fig. 2). However, since in this condition preferred item quantities and proportions were congruent, subjects’ performances cannot be taken as an unambiguous proof that monkeys made inferences solely based on food item proportions. Condition 3 controlled for such caveats, and subjects’ performance supports the idea that monkeys are able to solve the task without only relying on peanut quantities. Similarly, monkeys’ performance in condition 4, where bean quantities were the same in both populations, showed that the monkeys could succeed without avoiding the jar with the highest quantity of non-preferred items.

To our knowledge, our study is the first to both show such performance in a macaque species in an active paradigm choice testing statistical inferences abilities and control for quantity-based choice heuristics. Indeed, as a group, long-tailed macaques exclusively succeeded at making inferences when proportions were simply reversed (Placì et al., 2018). Contrary to Tonkean macaques, long-tailed macaques failed at drawing inferences when preferred or non-preferred item quantities remained the same between populations, but proportions varied. While capuchin monkeys presented more equivalent results to those of Tonkean macaques, as they made statistical inferences based on proportions (*i.e.,* when quantities and proportions were incongruent), they failed when non-preferred item quantities were the same in both populations (Tecwyn et al., 2017). Interestingly, when proportions and quantities were congruent (condition 1), Tonkean macaques achieved the task successfully with a performance rate of 84% whereas long-tailed macaques and capuchin monkeys reached a performance of around 64%, which was, although significant, only slightly above the level of chance. The higher performance rate of Tonkean macaques could be explained by methodological differences between studies. We implemented a longer training period with three consecutive sessions that subjects needed to validate before being tested. Discrepancy in training methods may have enabled Tonkean macaques to understand the task better than in previous studies. In addition, results of both studies involving capuchins and long-tailed macaques presented numerous side-biased individuals (Tecwyn et al., 2017; Placì et al., 2018) which could have led to decreased group performance, although Tecwyn et al. (2017) withdrew individuals that had a 100% side-biases from their analyses. In our study, we attempted to control and limit side-biases by assessing both whether an individual was motivated to participate in the task (even if it would voluntarily join the experimental room) and whether it had residual side bias from previous sessions.

Taken together, our results in conditions 1, 3 and 4 were consistent with two main interpretations which cannot be disentangled at group level. Tonkean macaques could have solved the task by relying on proportions and thus would be able to make intuitive statistical inferences from populations to samples. But because each condition was designed to control one effect at a time, although presented in a random order, Tonkean macaques could also have flexibly switched strategies between conditions. They could rely either on preferred item quantities (condition 1 and 4) or on non-preferred item quantities (condition 3) according to conditions. Their performance in remaining conditions 2 and 5 showed that preferred item quantities could have played a significant role in their decision making. In condition 2, where quantities and proportions were incongruent and both preferred and non-preferred item quantities varied, we replicated one of the experiments made in long-tailed macaques and apes (“exp6;” Rakoczy et al., 2014; “exp2a;” Placì et al., 2018). In this condition, performances of Tonkean macaques were in line with those of long-tailed macaques as they were not different from chance level, but lower than apes (Fig. 3). This suggests that they neither relied on proportions in those conditions nor applied one of the rules based on quantities of items. The control conditions for absolute quantities we added in our experimental protocol (conditions 5a and 5b), nonetheless allow us to interpret the response of Tonkean macaques as biased towards higher quantities of preferred items. When proportions did not differ between populations, but only in total item quantities, Tonkean macaques preferentially chose the sample selected from the jar containing the most preferred items. Model analyses with group-level data also suggested that both proportions and preferred food quantities had a significant role to predict the drawing outcome (Table S4).

Contrary to previous studies in non-human primates (Tecwyn et al., 2017; Placì et al., 2018; Eckert et al., 2018a), we aimed to investigate whether the decisional strategies used to solve the task were different among individuals. Because each condition was designed to test a single effect at a time, as noted above, success or failure in a given condition was critical for the overall interpretation of the results. Aggregating performance at the group level may obscure meaningful individual variations in responses across conditions. Our data analysis therefore enabled us to unravel individual differences in decision-making strategies that emerged when solving the task. Our main interpretation of the data was based on individual performances’ analysis condition-by-condition (Table 3). We combined results of the secondary model analyses of individuals’ decisional strategies (Tables S5 and S6) to better understand individual response.

Results converged toward different profiles of individuals’ decisional strategy. One subjects exclusively solved the task by relying on proportions (*i.e., Ficelle*; all analyses were consistent; Table 3 and Tables S5, S6). Six individuals used mainly and significantly preferred item quantities when making inferences (*i.e., Alaryc*, *Abricot*, *Eric*, *Nereis*, *Nema* and *Horus*). Indeed, *Horus* did not choose the sample from the favourable population significantly more than chance in any of the incongruent conditions. However, the above-chance-level performance of *Abricot* and *Eric* in incongruent condition 3, where preferred item quantities were equal in both populations, might suggest they could use proportions in some contexts. For two individuals (*i.e., Barnabé* and *Olli*), analyses suggested they used a WS/LS strategy (Table 4), which seemed to better explain their performance across conditions, as none of the decisional strategies (*i.e.,* Table 2) clearly appeared. *Walt*’s performances were not consistent with any of the decisional strategies tested above.

In all, both between and within individual variations underlay the use of diverse cognitive strategies depending on context. Our results showed that a majority of individuals either relied on quantities rather than proportions to make inferences from population to sample, or adopt an alternative WS/LS strategy to solve the task. Indeed, for each individual, a differential trade-off could exist between the cognitive costs of making statistical inferences and the benefits of the rewards obtained. Even if inferential reasoning based on quantities is detrimental in this experimental task, it may appear to be favourable or at least not damaging in other contexts. In the wild, such incongruity between proportions and quantities of food may not occur so often, which could explain the proportion of subjects showing simpler heuristics rules. Comparing and choosing from proportions is a complex and thus a costly capacity that involves considering relation between relations of two kinds of food, rather than simply comparing two quantities of one food item (Drucker, Rossa & Brannon, 2016; Watzek & Brosnan, 2018). Hence, using cognitive shortcuts or suboptimal decisional strategies may lead to negligible losses and a satisfying short-term gain (Watzek & Brosnan, 2018). In our study, the two individuals which solved the task by relying on the previous outcome did not even need to pay attention to the different populations presented. The WS/LS strategy has been shown to be an efficient strategy to flexibly adapt in a context of varying probabilities (Nowak & Sigmund, 1993; Buechel et al., 2018). Therefore, the sum of costs and benefits for the use of a complex cognitive capacity may result in a no net advantage for an individual (Healy, 2012; Boogert et al., 2018). This trade-off would explain that different cognitive strategies emerged, with subjects on the one hand opting for less costly cognitive solutions, such as comparing preferred item quantities or relying on other heuristics, and much more motivated subjects on the other, who optimised their chances to obtain food using more complex cognitive abilities, such as making statistical inferences. Motivation has been shown to positively affect cognitive performance in various cognitive tasks (Laland & Reader, 1999; Altschul et al., 2017; Rathke & Fischer, 2020) and often acts in tandem with personality, emotional arousal and social context (Morand-Ferron, Cole & Quinn, 2016; Boogert et al., 2018; De Petrillo & Rosati, 2021a). Even after limiting the effect of the subjects’ motivation levels through our protocol, motivation, being a transient factor that varies greatly over time, may have also played a role in this cost-benefit balance when making statistical inferences.

Additionally, some subjects in some of the conditions may have chosen the jar containing the larger quantity of preferred food without taking into account the relative proportions of each proposed jar, as this latter choice may request more inhibitory control (Völter et al., 2018; Hubbard & McCowan, 2025). Because both jars were visible during the whole testing, some individuals may have not been able to restrain their impulsive attraction for the highest quantity of preferred food. For example, in the incongruent condition 3, where both populations had equal quantities of preferred items, *Abricot* and *Eric* chose the sample from the favourable population in a high majority of trials, but mostly chose the sample from the population with the highest quantity of preferred items in incongruent conditions 2a and 2b, where preferred item quantities varied a lot between populations. In comparison, in the study testing intuitive statistical inferences in capuchin monkeys, subjects successfully passed an incongruent condition where the unfavourable population contained only twice as many preferred items as the other (Tecwyn et al., 2017). In conditions 2a and 2b of our task, the unfavourable populations presented eight times and three times as many preferred items as the other jar, respectively, which might have made the differences too large for some individuals to overcome their attraction to peanuts. A lack of inhibitory control in macaques could in the same way explain the difference in results with apes. Several studies showed that apes possess higher self-control capacities than several monkey species thanks to multiple inhibitory-control tasks (Amici, Aureli & Call, 2008; MacLean et al., 2014; Amici et al., 2018). Studies comparing different macaque species concluded that while Tonkean macaques had better inhibitory-control competences than less socially tolerant species (*i.e.,* rhesus macaques and to a lesser extent long-tailed macaques), they still showed poor performances in the various inhibitory-control tasks presented (Joly et al., 2017; Loyant et al., 2023).

In these same incongruent conditions 2a and 2b, although the proportions in the populations were identical, the total quantities of items varied, as our aim was to evaluate whether the total number of items could influence the perception of proportionality. It appeared that results between these conditions were not significantly different (Fig. 3). In fact, a study in angelfish (*Pterophyllum scalare*) showed that they discriminate quantities based on a continuous variable, namely shoal density, to estimate which shoal was larger (Gómez-Laplaza & Gerlai, 2013). In the same way, macaques in the study of Drucker and collaborators (2016) discriminated proportions by relying on both numerical ratio and stimuli area ratio. In our experiment, Tonkean macaques may have discriminated proportions and quantities by relying on the colour density of the items visible through the jars. Our aim was not to determine which perceptual cues macaques relied on to discriminate quantities or proportions. However, it would be interesting for future studies to investigate which continuous quantitative cues are used and how they could influence proportions’ discrimination and, consequently, intuitive statistical inferences.

One limitation of our study was that we did not vary the ROR values extensively, which could have helped to better disentangle decision-making strategies across conditions (as in analysis Table S5) and allowed a more direct comparison with results obtained in apes (Eckert et al., 2018a). In a study with apes, Eckert and collaborators demonstrated that performance at making intuitive inferences depended on the ratio of proportions that needed to be discriminated, according to Weber’s law (Eckert et al., 2018a) which postulates that the ability to discriminate between two stimuli depends on their proportional (or relative) differences in magnitude rather than their absolute differences (Fechner, 1965; Bullough et al., 2023). In their study, a ROR of 16 corresponded to the easiest proportion apes could discriminate. As our goal was to detect statistical inferences capacities, we chose to keep ROR constant across test conditions. In a similar study to ours, capuchin monkeys failed at drawing inferences from two populations that presented the same quantities of non-preferred items but differed in proportions (Tecwyn et al., 2017). In their task, the ROR was just above 4 which corresponded to a mean proportion of correct choices of 53,1% in chimpanzees, not different from chance level. This low ROR could then explain the poor results of capuchins. In our study, we thus designed our experimental conditions by choosing in all test conditions the most extreme ROR that apes could discriminate, that was 16. Only few studies investigated the applicability of Weber’s law in proportion discrimination (Honig & Stewart, 1989; Emmerton, 2001; Vallentin & Nieder, 2008; Drucker, Rossa & Brannon, 2016; Roberts, MacDonald & Lo, 2018) and even less so in a context of intuitive statistics (Eckert et al., 2018a; Johnston, Brecht & Nieder, 2023). Further work should thus vary ROR not only to better understand the applicability of Weber’s law in intuitive statistics in monkeys, but also to be able to better analyse the use of proportions in making inferences.

## Conclusion

To conclude, our work provides evidence that at least one Tonkean macaque was able to make intuitive statistical inferences. However, most of the macaques solved the task by combining several decision-making strategies based on preferred item quantities and the reward obtained in the previous trial (*i.e.,* win stay/lose shift strategy). Recent studies on non-primate species showed some intuitive statistical inferences abilities in keas (*Nestor notabilis*: Bastos & Taylor, 2020) and giraffes (*Giraffa camelopardalis*: Caicoya, Colell & Amici, 2023). However, given the few numbers of studies, it seems premature to conclude about whether intuitive probabilistic inference skills are shared beyond primate taxa or result from an evolutionary convergence (Nieder, 2018; De Petrillo & Rosati, 2019a). Given the extent to which making intuitive statistical inferences can apply across multiple other cognitive challenges, and our encouraging results, future studies should continue to explore intuitive statistical inferences in monkey species, to further determine their ability to rely on proportions (*e.g.*, variations of ROR) or other heuristics (*e.g.*, individual-level analyses) to make inferences, as well as the limits of this capacity.

##  Supplemental Information

10.7717/peerj.21377/supp-1 Supplemental Information 1 Effect of adding a dried grape on performance in test conditions Number of trials in which inferences were made from the favourable jar (in green) or from the unfavourable jar (in red) is plotted against whether or not a dried grape was given in addition to a peanut. Five individuals (*Eric*, *Ficelle*, *Horus*, *Olli* and *Walt*) are included in these analyses as they were the only ones to have carried out some sessions without and with a dried grape in certain conditions excluding control conditions: four individuals are included in condition 1, 3 and 2a but only two individuals are included in condition 4 analyses.

10.7717/peerj.21377/supp-2 Supplemental Information 2 Schematic representation of the ten positions taken by the experimenter In each position, the experimenter places himself in such a way that he never had visual access to the jars when he picked the items. The arrow shows the orientation of the head and the eyes (which were closed). The ten positions shown in the diagram are typical positions designed to provide the experimenter with reference points but were in no way stereotyped (each posture assumed by the experimenter varied from one trial to another for the same typical position). They were performed in a random order within a session.

10.7717/peerj.21377/supp-3 Supplemental Information 3 Decision tree for side-bias management For each test session in experimental conditions, the presence or absence of a side-bias was determined with this decision tree. If the same side was chosen in 80% of trials (8/10 trials) in one session, a side bias was detected (grey box with red outline). When the same side was chosen in less than 80% of trials, we only considered the crossed-arms trials. Indeed, an individual could always choose the same side but could have followed the arms when they were crossed which could have led to a false result of 50% of each side chosen. A side-bias was detected when the same side was chosen in 100% of the arm-crossed trials (grey box with red outline). To counter-balance side-biases, we conducted two visible-item sessions. If no mistake was made at these two sessions, subjects continued with training condition sessions. When individuals were already in the experimental conditions testing phase, they needed to made less than 2 mistakes in two consecutive training condition sessions, to come back to experimental condition sessions.

10.7717/peerj.21377/supp-4 Supplemental Information 4 Decision tree for subject’s inclusion in test trials Two phases of the experimental procedure are described (grey boxes): training condition and experimental conditions. After passing the training condition (green box), subjects started the experimental conditions. At the beginning of each test session, four motivation trials were conducted to check for subject’s motivation and absence of side-biases. Results of these motivation trials were divided into three cases (blue boxes) which led to different outcomes depending on whether or not a side-bias had been detected during the previous test session. If the subject made no mistake on the four trials, it was tested (green box). If the subject made more than one mistake, it was not tested (red outline box) but either the session was delayed (absence of side-bias) or subject came back to the training condition (presence of side-bias). If exactly one mistake was made, a presence of a side-bias during the previous session led to a comeback at the training condition (red outline box). In case of an absence of side-bias, four supplementary motivation trials were conducted (grey box) and the subject was tested (green box) if no mistake was made at these new motivation trials.

10.7717/peerj.21377/supp-5 Supplemental Information 5 Correlation between group performance and trial number for each condition The dark lines indicate a linear regression model using the Pearson correlation coefficient (R) to assess the linear relationship between the number of trials and the performance in each condition. The light lines indicate the closest fit to the data points. In conditions 5a, 2b and 5b monkeys tend to increase their choice for the fullest jar when number of trials increases in these conditions.

10.7717/peerj.21377/supp-6 Supplemental Information 6 List of individuals which were given a dried grape each time they obtained a peanut Thanks to the four motivational trials made before starting test trials, we were able to detect at an early stage, a lack of motivation for peanuts for some individuals. Some individuals never get a dried grape (“None” column “Condition” and “Start point”), some others had this protocol since the beginning of the testing procedure (“Training” column “Start point”) and others started having a dried grape in the middle of the procedure. The column ”Start point” corresponds to the session from which individuals were given a dried grape each time they obtained a peanut. From the moment we added a raisin to the peanuts for a particular subject, we never went back and kept doing it until the end of all sessions. For all sessions before the start point, individuals obtained solely a peanut, for all session after this starting point, individuals were given a dried grape when they obtained a peanut. The column ”Number of sessions” indicates the number of sessions in which individuals were given a dried grape when they obtained a peanut. The column ”Conditions” indicate at which condition individuals were given a dried grape when they obtained a peanut, in bold two sessions of the same condition.

10.7717/peerj.21377/supp-7 Supplemental Information 7 Effect on performance of giving a dried grape in addition to the peanut obtained The effect of getting a dried grape after obtaining a peanut was evaluated by chi-square tests in six conditions. Five individuals (*Eric*, *Ficelle*, *Horus*, *Olli* and *Walt*) were included in these analyses as they were the only ones to have carried out some sessions without and with a dried grape in certain conditions (six conditions). No significant difference was observed (all p¿0.1).

10.7717/peerj.21377/supp-8 Supplemental Information 8 Weber’s law testing model analyses Weber’s law posits that the capacity to discriminate two stimuli on the basis of their magnitudes, depends on the ratio of these magnitudes rather than their absolute differences (Bullough et al., 2023; Fechner, 1965) . To check whether monkeys based their reasoning on Weber’s law, we investigated how the monkey’s choice was affected by either the absolute difference in peanuts’ quantity or the ratio of peanuts’ quantity between two jars. In the following section, we will use the notation QP to express the quantity of peanuts in one jar. We fitted two different generalised linear models (GLM) with a binomial distribution and a logit link function (model (a) and model (b)). The first model fitted significantly to the data with an AIC of 1960.4. The second model fitted significantly to the data with an AIC of 1903.4. This analysis confirmed that models expressing peanuts quantities difference between two jars of a same condition using a ratio of quantities better fitted the decisions of the subjects. Therefore, our data are consistent with literature and follow Weber’s law. In the following models, we thus formulate the difference in items quantities between jars as a ratio of quantities.

10.7717/peerj.21377/supp-9 Supplemental Information 9 Model selection for group data GLMM analyses We ran several GLMM models to investigate whether monkeys were relying on peanuts proportions (ROR), peanuts quantities (RQP) or beans quantities (RQB) to infer the nature of a single item randomly sampled from different populations across all conditions (excluded olfactory control condition). The four GLMM had a binomial distribution and a logit link function. We put the identity of individuals and the position of the experimenter (see supplementary materials Figure S2). We compared these models based on the BIC criterion and the “Variance Inflated Factor” (VIF). Model (A) had a too high VIF ranging between 3 and 6 for our variable of interests (RQP, RQB and ROR). In the three models left, these variables presented a VIF between 1 and 2. In all models, we evaluated the effect of experimental factors that varied during the task other than food items quantities. Model (B) had the lowest BIC and valid VIF values which makes it the best-fitting model for the group data.

10.7717/peerj.21377/supp-10 Supplemental Information 10 Individual quantity-based or proportion-based strategies after model selection The results of the models that best fit each individual’s behaviour are presented in this table. Each model is a GLM with a binomial family and a logit link function applied to individual-level data. The fixed variable was the jar chosen and the explanatory variables were the logarithm of the ratio of peanuts quantities between jars (RQP), the ratio of peanuts proportions between jars (ROR) or the ratio of beans quantities between jars (RQB), depending on the model selected for each individual. When BIC values differed by less than two points between several models for one individual, the results of all these models are presented (e.g. *Barnabé*). * indicates significant p-values.

10.7717/peerj.21377/supp-11 Supplemental Information 11 First trial performance across conditions For each condition, the table reports the number of successes (“#Success”), the total number of individuals (“#Total”), and the corresponding performance (“Perf”). Differences between first-trial performance and chance level (0.5) were tested using two-tailed binomial tests. P-values are reported for each condition.

10.7717/peerj.21377/supp-12 Supplemental Information 12 R-Code

10.7717/peerj.21377/supp-13 Supplemental Information 13 Raw Data

10.7717/peerj.21377/supp-14 Supplemental Information 14 Data coding reliance dataset

## Additional Information and Declarations

### Data Availability

The following information was supplied regarding data availability: The raw data and code are available in the Supplemental Files. The raw data and code are available at Zenodo: - Beaud, A., Meunier, H., Ballesta, S., & Detourbet, A. (2026). Data - code - supplementary video repository. Zenodo. https://doi.org/10.5281/zenodo.19117706.
