# Supplementary figures and images for: Are Tonkean macaques able to make intuitive statistical inferences?
Source: PeerJ. 2026 Jun 30;14:e21377. doi: 10.7717/peerj.21377 (PMC13330748; doi:10.7717/peerj.21377)

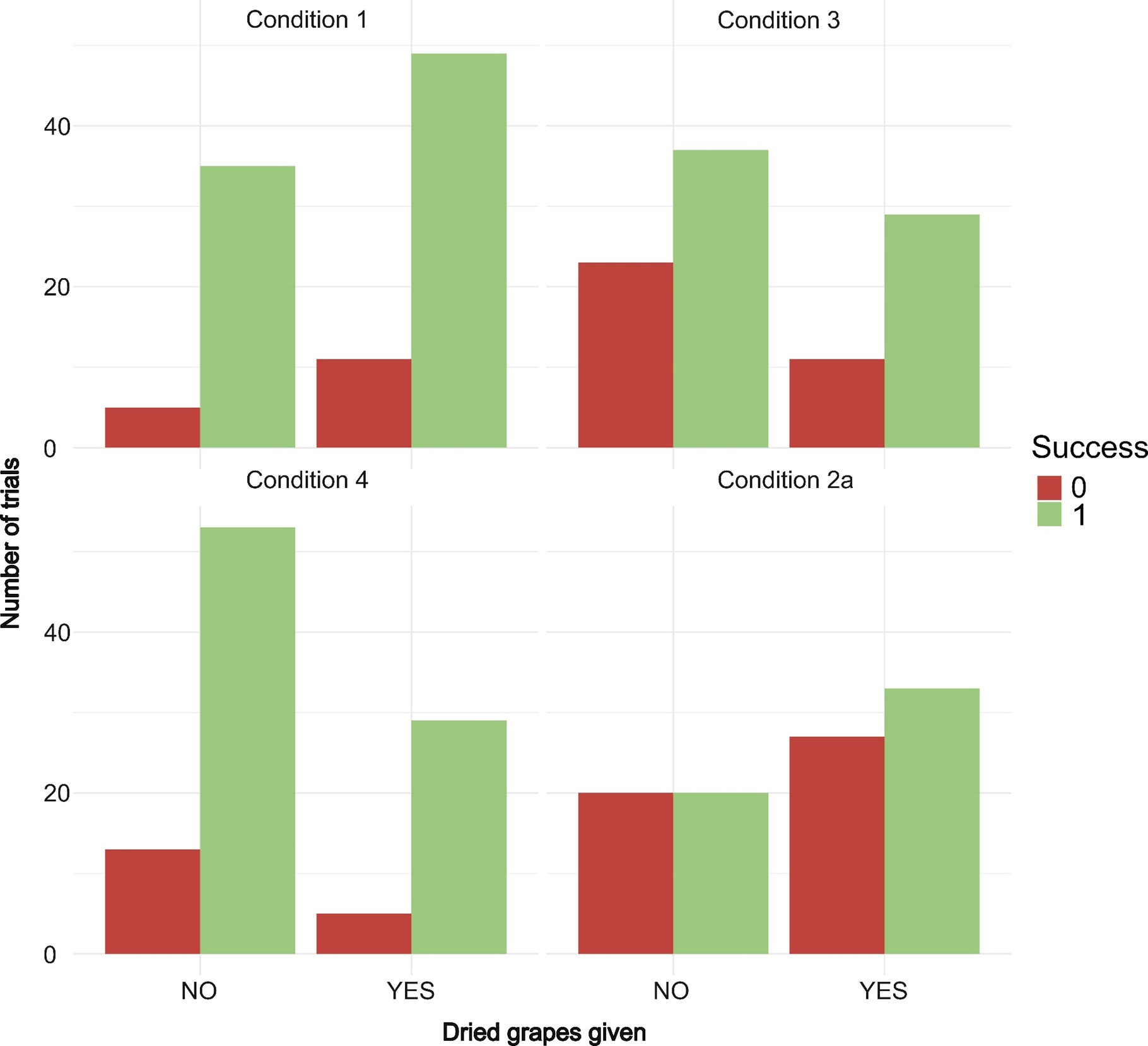

Supplement: Supplemental Information 1 — Number of trials in which inferences were made from the favourable jar (in green) or from the unfavourable jar (in red) is plotted against whether or not a dried grape was given in addition to a peanut. Five individuals (Eric, Ficelle, Horus, Olli and Walt) are included in these analyses as they were the only ones to have carried out some sessions without and with a dried grape in certain conditions excluding control conditions: four individuals are included in condition 1, 3 and 2a but only two individuals are included in condition 4 analyses. [file peerj-14-21377-s001.jpg]

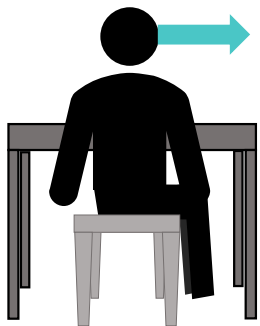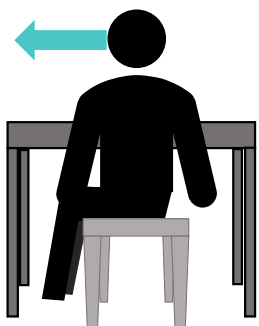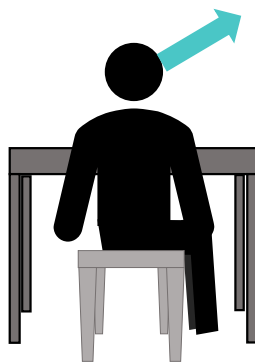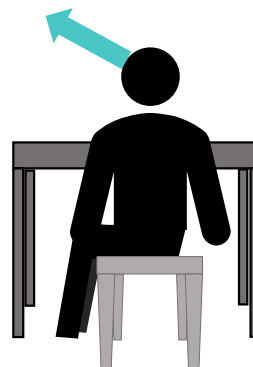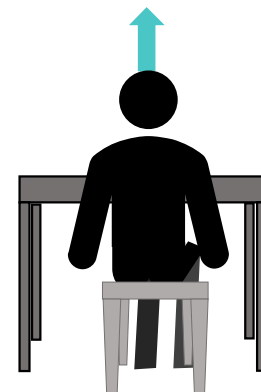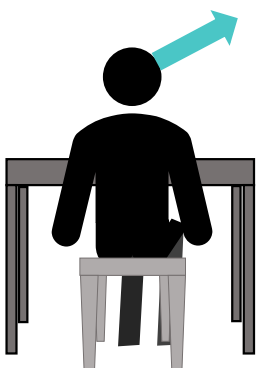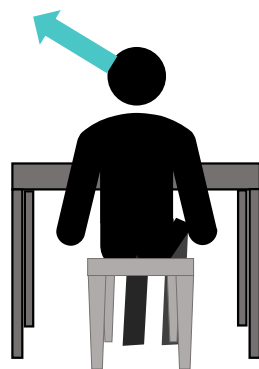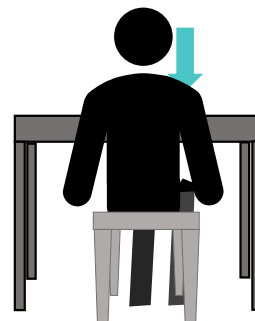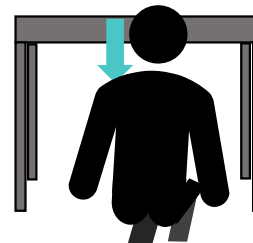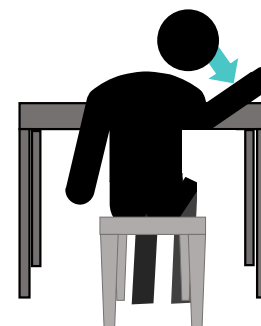

Supplement: Supplemental Information 2 — In each position, the experimenter places himself in such a way that he never had visual access to the jars when he picked the items. The arrow shows the orientation of the head and the eyes (which were closed). The ten positions shown in the diagram are typical positions designed to provide the experimenter with reference points but were in no way stereotyped (each posture assumed by the experimenter varied from one trial to another for the same typical position). They were performed in a random order within a session. [file peerj-14-21377-s002.pdf]

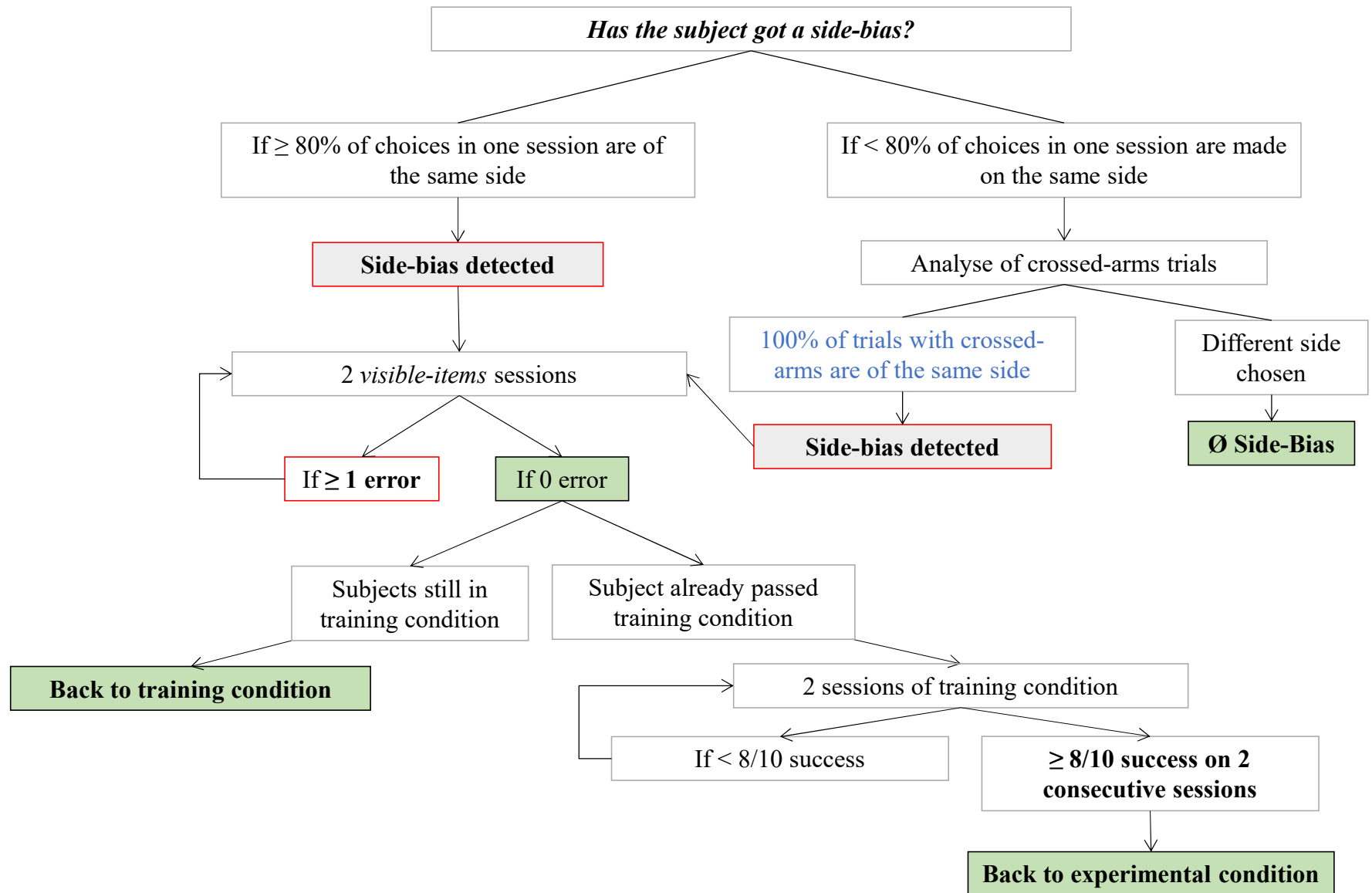

Supplement: Supplemental Information 3 — For each test session in experimental conditions, the presence or absence of a side-bias was determined with this decision tree. If the same side was chosen in 80% of trials (8/10 trials) in one session, a side bias was detected (grey box with red outline). When the same side was chosen in less than 80% of trials, we only considered the crossed-arms trials. Indeed, an individual could always choose the same side but could have followed the arms when they were crossed which could have led to a false result of 50% of each side chosen. A side-bias was detected when the same side was chosen in 100% of the arm-crossed trials (grey box with red outline). To counter-balance side-biases, we conducted two visible-item sessions. If no mistake was made at these two sessions, subjects continued with training condition sessions. When individuals were already in the experimental conditions testing phase, they needed to made less than 2 mistakes in two consecutive training condition sessions, to come back to experimental condition sessions. [file peerj-14-21377-s003.pdf]

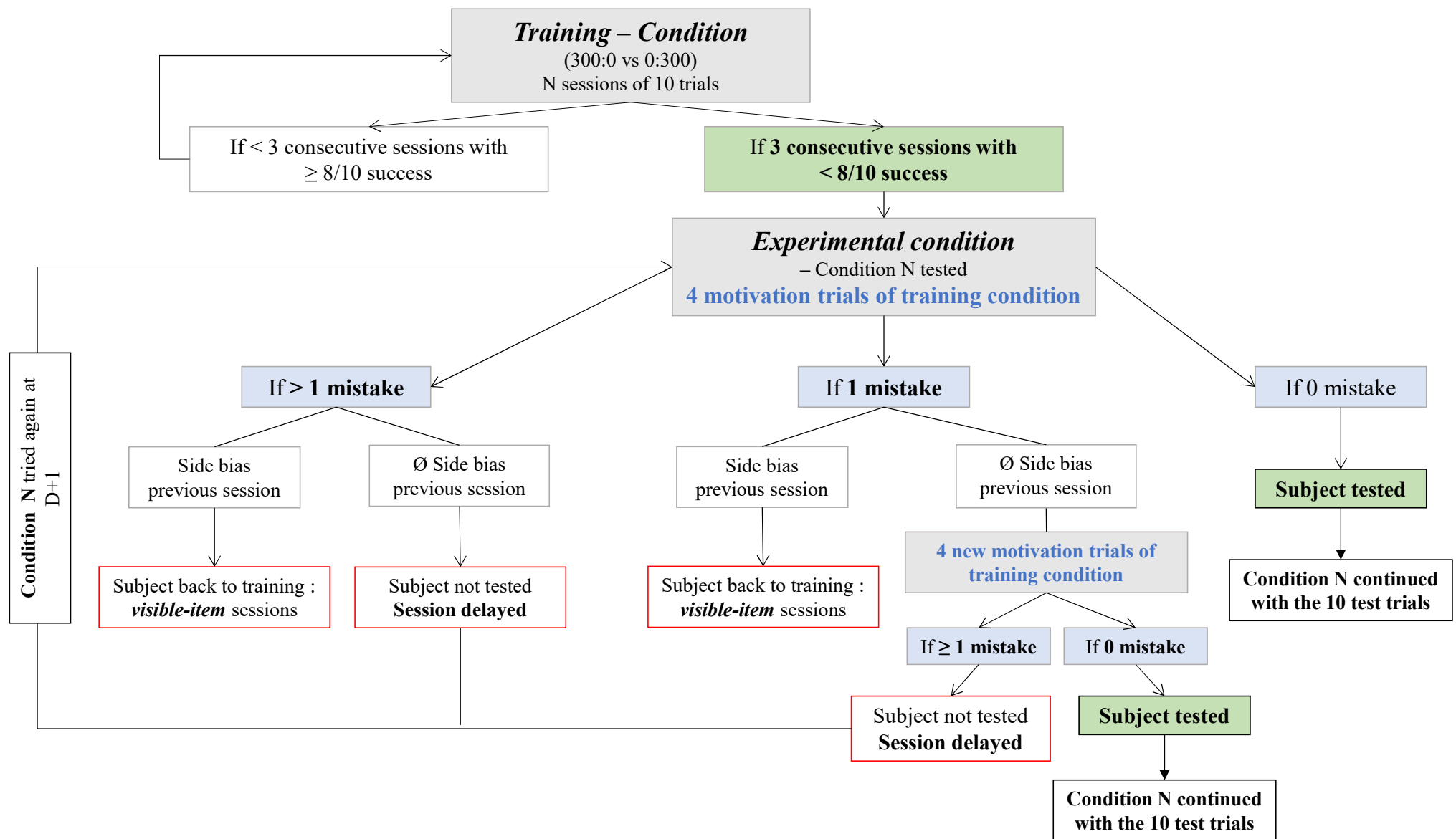

Supplement: Supplemental Information 4 — Two phases of the experimental procedure are described (grey boxes): training condition and experimental conditions. After passing the training condition (green box), subjects started the experimental conditions. At the beginning of each test session, four motivation trials were conducted to check for subject’s motivation and absence of side-biases. Results of these motivation trials were divided into three cases (blue boxes) which led to different outcomes depending on whether or not a side-bias had been detected during the previous test session. If the subject made no mistake on the four trials, it was tested (green box). If the subject made more than one mistake, it was not tested (red outline box) but either the session was delayed (absence of side-bias) or subject came back to the training condition (presence of side-bias). If exactly one mistake was made, a presence of a side-bias during the previous session led to a comeback at the training condition (red outline box). In case of an absence of side-bias, four supplementary motivation trials were conducted (grey box) and the subject was tested (green box) if no mistake was made at these new motivation trials. [file peerj-14-21377-s004.pdf]

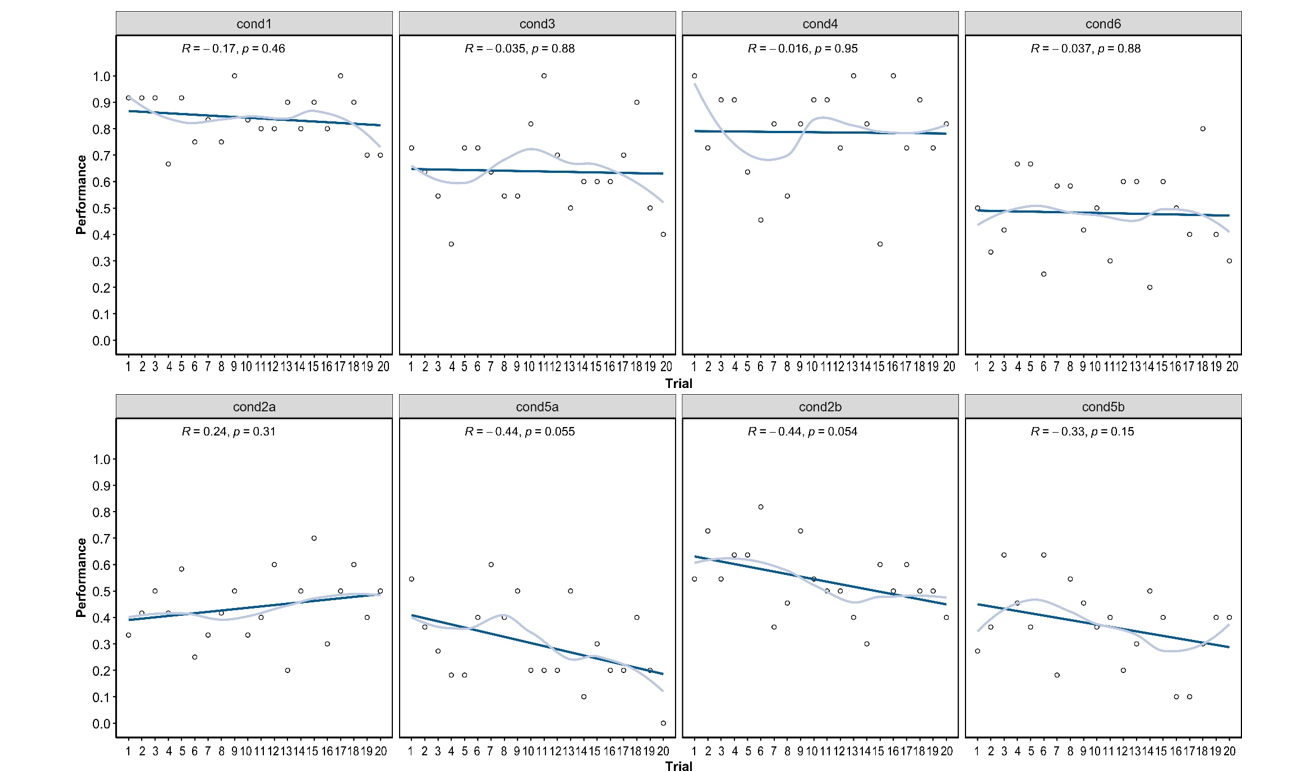

Supplement: Supplemental Information 5 — The dark lines indicate a linear regression model using the Pearson correlation coefficient (R) to assess the linear relationship between the number of trials and the performance in each condition. The light lines indicate the closest fit to the data points. In conditions 5a, 2b and 5b monkeys tend to increase their choice for the fullest jar when number of trials increases in these conditions. [file peerj-14-21377-s005.png]
