# Supplementary material for: Are Tonkean macaques able to make intuitive statistical inferences?
Source: PeerJ. 2026 Jun 30;14:e21377. doi: 10.7717/peerj.21377 (PMC13330748; doi:10.7717/peerj.21377)
Supplement: Supplemental Information 6 — Thanks to the four motivational trials made before starting test trials, we were able to detect at an early stage, a lack of motivation for peanuts for some individuals. Some individuals never get a dried grape (“None” column “Condition” and “Start point”), some others had this protocol since the beginning of the testing procedure (“Training” column “Start point”) and others started having a dried grape in the middle of the procedure. The column ”Start point” corresponds to the session from which individuals were given a dried grape each time they obtained a peanut. From the moment we added a raisin to the peanuts for a particular subject, we never went back and kept doing it until the end of all sessions. For all sessions before the start point, individuals obtained solely a peanut, for all session after this starting point, individuals were given a dried grape when they obtained a peanut. The column ”Number of sessions” indicates the number of sessions in which individuals were given a dried grape when they obtained a peanut. The column ”Conditions” indicate at which condition individuals were given a dried grape when they obtained a peanut, in bold two sessions of the same condition. [file peerj-14-21377-s006.docx]

| **Individual** | **Start point** | **Number of sessions** | **Conditions** |
| --- | --- | --- | --- |
| Abricot | Training | 16/16 | **1-2a-2b-3-4-5a-5b-6** |
| Alaryc | Training | 16/16 | **1-2a-2b-3-4-5a-5b-6** |
| Barnabe | Training | 16/16 | **1-2a-2b-3-4-5a-5b-6** |
| Dory | 4e session | 1/4 | **2b** |
| Eric | 8e session | 9/16 | 1-2a-**2b**-3-**4**-5a-5b |
| Ficelle | 9e session | 8/16 | 1-2a-**2b**-3-4-**5b** |
| Horus | 8e session | 9/16 | 1**-2a**-**2b**-3-5a-5b-6 |
| Jeanne | Training | 4/4 | 1-2a-3-5b |
| Nema | Training | 16/16 | **1-2a-2b-3-4-5a-5b-6** |
| Nereis | None | 0/16 | None |
| Olli | 9e session | 8/16 | **1**-**2b**-2a-5a-5b-6 |
| Wallace | None | 0/4 | None |
| Walt | 10e session | 7/16 | 1-2a-**2b**-3-5a-5b |
