# Supplementary material for: Are Tonkean macaques able to make intuitive statistical inferences?
Source: PeerJ. 2026 Jun 30;14:e21377. doi: 10.7717/peerj.21377 (PMC13330748; doi:10.7717/peerj.21377)
Supplement: Supplemental Information 7 — The effect of getting a dried grape after obtaining a peanut was evaluated by chi-square tests in six conditions. Five individuals (Eric, Ficelle, Horus, Olli and Walt) were included in these analyses as they were the only ones to have carried out some sessions without and with a dried grape in certain conditions (six conditions). No significant difference was observed (all p¿0.1). [file peerj-14-21377-s007.docx]

| **Condition** | ***X-square*** | ***p*** |
| --- | --- | --- |
| **1** | 0.25 | 0.62 |
| **2a** | 0.08 | 0.77 |
| **3** | 0.82 | 0.37 |
| **4** | 0.12 | 0.73 |
| **5a** | 1.68 | 0.19 |
| **5b** | 0.49 | 0.48 |
