# Supplementary material for: Are Tonkean macaques able to make intuitive statistical inferences?
Source: PeerJ. 2026 Jun 30;14:e21377. doi: 10.7717/peerj.21377 (PMC13330748; doi:10.7717/peerj.21377)
Supplement: Supplemental Information 8 — Weber’s law posits that the capacity to discriminate two stimuli on the basis of their magnitudes, depends on the ratio of these magnitudes rather than their absolute differences (Bullough et al., 2023; Fechner, 1965) . To check whether monkeys based their reasoning on Weber’s law, we investigated how the monkey’s choice was affected by either the absolute difference in peanuts’ quantity or the ratio of peanuts’ quantity between two jars. In the following section, we will use the notation QP to express the quantity of peanuts in one jar. We fitted two different generalised linear models (GLM) with a binomial distribution and a logit link function (model (a) and model (b)). The first model fitted significantly to the data with an AIC of 1960.4. The second model fitted significantly to the data with an AIC of 1903.4. This analysis confirmed that models expressing peanuts quantities difference between two jars of a same condition using a ratio of quantities better fitted the decisions of the subjects. Therefore, our data are consistent with literature and follow Weber’s law. In the following models, we thus formulate the difference in items quantities between jars as a ratio of quantities. [file peerj-14-21377-s008.docx]

|  | Model (a) | | | | Model (b) | | | |
| --- | --- | --- | --- | --- | --- | --- | --- | --- |
|  | **Choice for the highest QP** | | | | **Choice for the highest QP** | | | |
| *Predictors* | *Estimates* | *SE* | *Statistic* | *p* | *Estimates* | *SE* | *Statistic* | *p* |
| (Intercept) | 0.17 | 0.08 | 2.06 | **0.039** | 1.17 | 0.09 | 12.41 | **<0.001** |
|  | 0.002 | 0.0003 | 4.93 | **<0.001** |  |  |  |  |
| $Higher QP -Lower QP$ |  |  |  |  | -3.38 | 0.38 | -8.88 | **<0.001** |
| AIC  $\frac{Lower QP}{QP jar A+QP jar B}$ | 1960.394 | | | | 1903.355 | | | |
