# Supplementary material for: Are Tonkean macaques able to make intuitive statistical inferences?
Source: PeerJ. 2026 Jun 30;14:e21377. doi: 10.7717/peerj.21377 (PMC13330748; doi:10.7717/peerj.21377)
Supplement: Supplemental Information 9 — We ran several GLMM models to investigate whether monkeys were relying on peanuts proportions (ROR), peanuts quantities (RQP) or beans quantities (RQB) to infer the nature of a single item randomly sampled from different populations across all conditions (excluded olfactory control condition). The four GLMM had a binomial distribution and a logit link function. We put the identity of individuals and the position of the experimenter (see supplementary materials Figure S2). We compared these models based on the BIC criterion and the “Variance Inflated Factor” (VIF). Model (A) had a too high VIF ranging between 3 and 6 for our variable of interests (RQP, RQB and ROR). In the three models left, these variables presented a VIF between 1 and 2. In all models, we evaluated the effect of experimental factors that varied during the task other than food items quantities. Model (B) had the lowest BIC and valid VIF values which makes it the best-fitting model for the group data. [file peerj-14-21377-s009.docx]

|  | **Model (A)** | | | | **Model (B)** | | | |
| --- | --- | --- | --- | --- | --- | --- | --- | --- |
| *Predictors* | *Estimates* | *SE* | *Statistic* | *p* | *Estimates* | *SE* | *Statistic* | *p* |
| (Intercept) | -0.02 | 0.14 | -0.16 | 0.875 | -0.03 | 0.14 | -0.19 | 0.851 |
| logRQP | 0.61 | 0.10 | 5.87 | **<0.001** | 0.66 | 0.06 | 10.77 | **<0.001** |
| logRQB | 0.07 | 0.14 | 0.51 | 0.610 |  |  |  |  |
| logROR | 0.56 | 0.11 | 4.90 | **<0.001** | 0.51 | 0.06 | 9.04 | **<0.001** |
| Arms position [1] | 0.02 | 0.11 | 0.16 | 0.869 | 0.02 | 0.11 | 0.16 | 0.870 |
| Fav Jar position [G] | -0.05 | 0.11 | -0.42 | 0.676 | -0.05 | 0.11 | -0.42 | 0.676 |
| Experimenter ID [2] | 0.18 | 0.14 | 1.28 | 0.202 | 0.18 | 0.14 | 1.29 | 0.199 |
| Session [2] | 0.05 | 0.12 | 0.46 | 0.645 | 0.05 | 0.12 | 0.47 | 0.638 |
| BIC | 1908.9 | | | | 1901.9 | | | |
|  | **Model (C)** | | | | **Model (D)** | | | |
| *Predictors* | *Estimates* | *SE* | *Statistic* | *p* | *Estimates* | *SE* | *Statistic* | *p* |
| (Intercept) | 0.03 | 0.14 | 0.18 | 0.854 | -0.06 | 0.14 | -0.38 | 0.701 |
| logRQP |  |  |  |  | 0.98 | 0.08 | 12.87 | **<0.001** |
| logRQB | 0.75 | 0.08 | 9.39 | **<0.001** | -0.52 | 0.07 | -7.59 | **<0.001** |
| logROR | 1.07 | 0.08 | 12.89 | **<0.001** |  |  |  |  |
| Arms position [1] | 0.02 | 0.11 | 0.19 | 0.850 | 0.02 | 0.11 | 0.15 | 0.884 |
| Fav Jar position [G] | -0.05 | 0.11 | -0.44 | 0.662 | -0.05 | 0.11 | -0.41 | 0.682 |
| Experimenter ID [2] | 0.15 | 0.14 | 1.08 | 0.280 | 0.19 | 0.14 | 1.38 | 0.167 |
| Session [2] | 0.04 | 0.11 | 0.33 | 0.738 | 0.06 | 0.11 | 0.53 | 0.595 |
| BIC | 1938.1 | | | | 1926.5 | | | |
