# Supplementary material for: Are Tonkean macaques able to make intuitive statistical inferences?
Source: PeerJ. 2026 Jun 30;14:e21377. doi: 10.7717/peerj.21377 (PMC13330748; doi:10.7717/peerj.21377)
Supplement: Supplemental Information 10 — The results of the models that best fit each individual’s behaviour are presented in this table. Each model is a GLM with a binomial family and a logit link function applied to individual-level data. The fixed variable was the jar chosen and the explanatory variables were the logarithm of the ratio of peanuts quantities between jars (RQP), the ratio of peanuts proportions between jars (ROR) or the ratio of beans quantities between jars (RQB), depending on the model selected for each individual. When BIC values differed by less than two points between several models for one individual, the results of all these models are presented (e.g. Barnabé). * indicates significant p-values. [file peerj-14-21377-s010.docx]

| **Model (7)** | **Intercept** | | | **LogROR** | | | **BIC** |  |  |  |
| --- | --- | --- | --- | --- | --- | --- | --- | --- | --- | --- |
| Individuals | Estimates | *SE* | *p* | Estimates | *SE* | *p* |  |  |  |  |
| **Ficelle** | -0,18 | 0,19 | 0,34 | 1,08 | 0,21 | **< 0,01*** | 169,8 |  |  |  |
| **Olli** | 0,00 | 0,18 | 1,00 | 0,89 | 0,19 | **< 0,01*** | 179,9 |  |  |  |
| **Walt** | -0,07 | 0,19 | 0,71 | 0,98 | 0,20 | **< 0,01*** | 175,4 |  |  |  |
| **Barnabé** | 0,06 | 0,18 | 0,72 | 0,68 | 0,18 | **< 0,01*** | 189,0 |  |  |  |
|  |  |  |  |  |  |  |  |  |  |  |
| **Model (5)** | **Intercept** | | | **LogRQP** | | | **BIC** |  |  |  |
| Individuals | Estimates | *SE* | *p* | Estimates | *SE* | *p* |  |  |  |  |
| **Alaryc** | -0,04 | 0,20 | 0,84 | 1,35 | 0,25 | **< 0,01*** | 162,2 |  |  |  |
| **Eric** | -0,47 | 0,28 | 0,09 | 2,61 | 0,42 | **< 0,01*** | 118,4 |  |  |  |
| **Abricot** | -0,11 | 0,20 | 0,59 | 1,30 | 0,25 | **< 0,01*** | 165,0 |  |  |  |
| **Nereis** | 0,75 | 0,21 | < 0,01 | 1,35 | 0,24 | **< 0,01*** | 146,7 |  |  |  |
| **Nema** | -0,18 | 0,19 | 0,34 | 0,92 | 0,22 | **< 0,01*** | 180,8 |  |  |  |
|  |  |  |  |  |  |  |  |  |  |  |
| **Model (3)** | **Intercept** | | | **LogRQP** | | | **LogROR** | | | **BIC** |
| Individuals | Estimates | *SE* | *p* | Estimates | *SE* | *p* | Estimates | *SE* | *p* |  |
| **Horus** | 0,16 | 0,19 | 0,40 | 0,96 | 0,22 | **< 0,01*** | 0,5 | 0,19 | **< 0,01*** | 175,87 |
| **Barnabé** | 0,06 | 0,18 | 0,73 | 0,41 | 0,19 | **0,03*** | 0,7 | 0,19 | **< 0,01*** | 188,84 |
| **Nereis** | 0,76 | 0,22 | < 0,01 | 1,37 | 0,25 | **< 0,01*** | 0,4 | 0,21 | 0,07 | 148,16 |
| **Eric** | -0,51 | 0,29 | 0,07 | 2,73 | 0,45 | **< 0,01*** | 0,4 | 0,24 | 0,08 | 120,13 |
|  |  |  |  |  |  |  |  |  |  |  |
| **Model (2)** | **Intercept** | | | **LogRQP** | | | **LogRQB** | | | **BIC** |
| Individuals | Estimates | *SE* | *p* | Estimates | *SE* | *p* | Estimates | *SE* | *p* |  |
| **Barnabé** | 0,04 | 0,18 | 0,83 | 0,96 | 0,25 | **< 0,01*** | -0,9 | 0,24 | **< 0,01*** | 187,05 |
| **Eric** | -0,61 | 0,31 | 0,05 | 3,12 | 0,58 | **< 0,01*** | -0,5 | 0,33 | 0,12 | 120,71 |
