# Supplementary material for: Are Tonkean macaques able to make intuitive statistical inferences?
Source: PeerJ. 2026 Jun 30;14:e21377. doi: 10.7717/peerj.21377 (PMC13330748; doi:10.7717/peerj.21377)
Supplement: Supplemental Information 11 — For each condition, the table reports the number of successes (“#Success”), the total number of individuals (“#Total”), and the corresponding performance (“Perf”). Differences between first-trial performance and chance level (0.5) were tested using two-tailed binomial tests. P-values are reported for each condition. [file peerj-14-21377-s011.docx]

| **Conditions** | **#Success** | **#Total** | **Perf** | ***p*** |
| --- | --- | --- | --- | --- |
| **1** | 11 | 12 | 0,92 | 0,006 |
| **3** | 8 | 11 | 0,73 | 0,227 |
| **4** | 11 | 11 | 1,00 | 0,001 |
| **6** | 6 | 12 | 0,50 | 1,000 |
| **2a** | 4 | 12 | 0,33 | 0,388 |
| **5a** | 6 | 11 | 0,55 | 1,000 |
| **2b** | 6 | 11 | 0,55 | 1,000 |
| **5b** | 3 | 11 | 0,27 | 0,227 |
